# Differential biosynthesis and cellular permeability explain longitudinal gibberellin gradients in growing roots

**DOI:** 10.1073/pnas.1921960118

**Published:** 2021-02-18

**Authors:** Annalisa Rizza, Bijun Tang, Claire E. Stanley, Guido Grossmann, Markus R. Owen, Leah R. Band, Alexander M. Jones

**Affiliations:** ^a^Sainsbury Laboratory, Cambridge University, Cambridge CB2 1LR, United Kingdom;; ^b^Institute for Chemical and Bioengineering, Department of Chemistry and Applied Biosciences, ETH Zürich, 8093 Zürich, Switzerland;; ^c^Agroecology and Environment Research Division, Agroscope, 8046 Zürich, Switzerland;; ^d^Centre for Organismal Studies, Heidelberg University, 69120 Heidelberg, Germany;; ^e^Centre for Mathematical Medicine and Biology, School of Mathematical Sciences, University of Nottingham, Nottingham NG7 2RD, United Kingdom;; ^f^Division of Plant and Crop Sciences, School of Biosciences, University of Nottingham, Sutton Bonington LE12 5RD, United Kingdom

**Keywords:** gibberellin, hormone biosensor, cell growth, root development, mathematical modeling

## Abstract

Control over cell growth by mobile regulators underlies much of eukaryotic morphogenesis. In plant roots, cell division and elongation are separated into distinct longitudinal zones and both division and elongation are influenced by the growth regulatory hormone gibberellin (GA). Previously, a multicellular mathematical model predicted a GA maximum at the border of the meristematic and elongation zones. However, GA in roots was recently measured using a genetically encoded fluorescent biosensor, nlsGPS1, and found to be low in the meristematic zone grading to a maximum at the end of the elongation zone. Furthermore, the accumulation rate of exogenous GA was also found to be higher in the elongation zone. It was still unknown which biochemical activities were responsible for these mobile small molecule gradients and whether the spatiotemporal correlation between GA levels and cell length is important for root cell division and elongation patterns. Using a mathematical modeling approach in combination with high-resolution GA measurements in vivo, we now show how differentials in several biosynthetic enzyme steps contribute to the endogenous GA gradient and how differential cellular permeability contributes to an accumulation gradient of exogenous GA. We also analyzed the effects of altered GA distribution in roots and did not find significant phenotypes resulting from increased GA levels or signaling. We did find a substantial temporal delay between complementation of GA distribution and cell division and elongation phenotypes in a GA deficient mutant. Together, our results provide models of how GA gradients are directed and in turn direct root growth.

## Significance

Growth hormones are mobile chemicals that exert considerable influence over how multicellular organisms like animals and plants take on their shape and form. Of particular interest is the distribution of such hormones across cells and tissues. In plants, one of these hormones, gibberellin (GA), is known to regulate cell multiplication and cell expansion to increase the rate at which roots grow. In this work, biosensor measurements were combined with theoretical models to elucidate the biochemical mechanisms that direct GA distribution and how these patterns relate to root growth. Our detailed understanding of how GA distributions are controlled in roots should prove a valuable model for understanding the makings of the many other hormone distributions that influence how plants grow.

The location of cell division and expansion guided by mobile growth regulators is fundamental to morphogenesis in multicellular organisms. How such regulators are controlled and the extent to which regulator patterns influence cell growth patterns has received considerable interest. In plants, the hormone gibberellin (GA) is a mobile growth regulator that plays an essential role during multiple stages of the plant life cycle (from seed germination to reproductive development) and acts through the destabilization of growth repressive DELLA proteins (1–9). In *Arabidopsis thaliana* roots, where cell division and expansion are separated in distinct longitudinal zones, GA plays a key role in regulating both cell division in the meristematic zone (10) and cell elongation in the elongation zone (11). Deficiencies in GA biosynthesis indeed result in reduced length of the meristematic zone and in reduced length of elongated mature cells (12). Using a genetically encoded fluorescent biosensor, nlsGPS1, we recently found that GA levels correlate with cell length in growing roots (13). Nonetheless, how this GA distribution is generated and the quantitative relationship between GA distribution and cell growth patterning remained unclear.

Plant hormones often coordinate the signal integration of environmental conditions into plant developmental programs and can be synthesized locally as well as distally from the site of action. Within cells, biosynthetic and catabolic enzymes in combination with transport activities control GA levels, and thus the differential spatiotemporal distribution of enzymatic and transport activities can all contribute to GA distributions that occur during plant development. GA biosynthesis proceeds in several steps with the final steps occurring in the cytoplasm, where the precursor GA_12_ is converted to GA_9_ by gibberellin 20-oxidase (GA20ox) enzymes and GA_9_ is converted into bioactive GA_4_ by gibberellin 3-oxidase (GA3ox) enzymes (9, 14). Previous works highlighted how the expression pattern of isozymes of GA20ox and GA3ox families, which partly overlapped in different cell types, organs, and developmental stages, might generate GA patterns (15, 16). GA can be disabled by enzymes belonging to gibberellin 2-oxidase (GA2ox), GA methyltransferase and CYP714A families (15, 17, 18). As with *Arabidopsis* GA biosynthetic genes, GA catabolic genes present partly overlapping expression patterns in various organs and developmental stages, likely influencing the GA distributions initiated by GA biosynthesis.

In recent years, several transmembrane transporters belonging to the nitrate transporter (NRT) 1/peptide transporter family (NPF) and two sugar transporters SWEET13 and SWEET14 have been identified as GA importers (19–22), but as yet no GA exporters have been identified. Because GA is a weak acid, an acid-trap mechanism might also accomplish GA import from the low pH apoplasm into higher pH cytoplasm separate from transmembrane transporter activities (23).

To understand in a quantitative manner the factors controlling longitudinal GA distribution in roots, we previously developed a multicellular mathematical model that predicted GA dynamics in the root elongation zone (12). The model revealed that the rapid cell expansion as cells traverse this zone results in hormone dilution that significantly affects cellular GA concentration. Assuming that GA biosynthesis occurs only in the meristem (and not the elongation zone) based on pAtGA20ox1-GUS staining (12), the model predicted that the GA dilution caused a reducing gradient of GA along the elongation zone (with high GA levels in the meristem grading to lower GA levels in the mature zone). These results did not agree with subsequent measurements using nlsGPS1, in which endogenous GA was found to grade from lower levels in the smaller cells of the meristematic zone to higher levels at the end of the elongation zone (13). Furthermore, treatment with exogenous GA showed faster accumulation of GA in the elongation zone compared to the meristematic zone, suggesting the presence of an accumulation gradient of exogenous GA, hereafter referred to as an exogenous-GA-generated gradient, that is independent of GA biosynthesis. This exogenous-GA-generated gradient was consistent with measurements of roots treated with exogenous fluorescein-labeled GAs, where the fluorescence of labeled GAs accumulated preferentially in the elongation zone (24).

By using nlsGPS1 live-imaging, genetic and biochemical perturbations, and multiscale modeling, we now show that a longitudinal differential in GA biosynthesis in *Arabidopsis* roots is responsible for shaping endogenous GA distribution and that a longitudinal differential in cellular permeability for GA is responsible for the exogenous-GA-generated gradient. We also used genetic and biochemical perturbations to investigate the functional relationship between GA gradients and cell elongation gradients. Interestingly, increasing GA levels or GA signaling does not affect cell growth patterning in *Arabidopsis* roots, indicating that a local dose–response relationship is unlikely. Thus, we propose a cooperative local dose–response relationship in which increasing GA concentrations in the elongation zone act in concert with other signals to direct cell elongation. However, we also demonstrate that complementation of GA deficiency phenotypes is substantially delayed compared with complementation of GA levels. Thus, a hysteretic relationship could instead be functioning in which GA influences cell elongation via prior signaling in the meristematic zone.

## Results and Discussion

### Gibberellin Biosynthesis Is Essential for Creating the GA Gradient in *Arabidopsis* Roots.

**Fig. 1.**
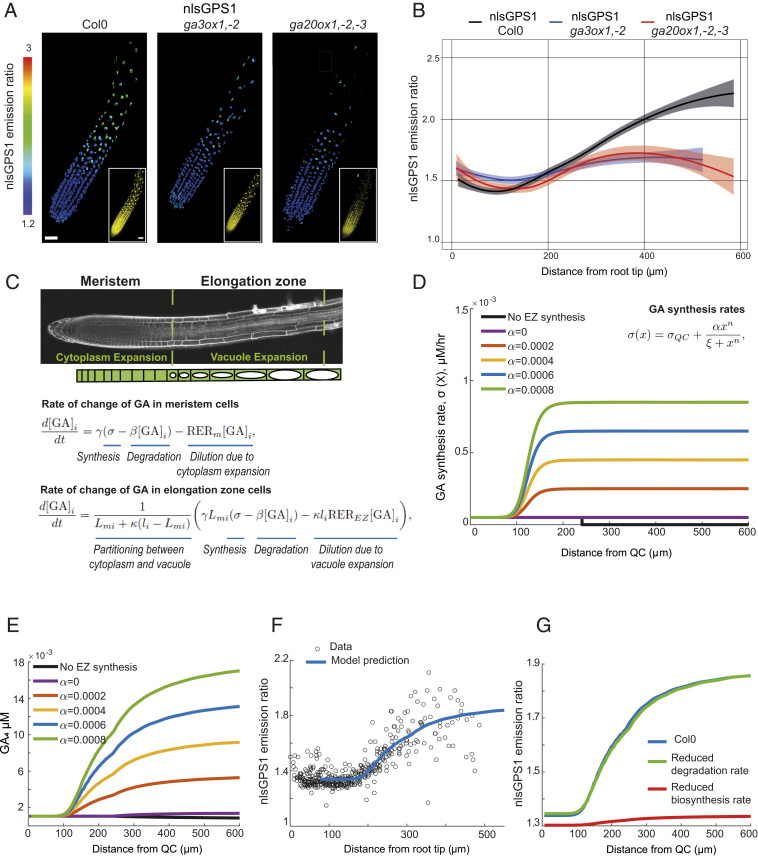
GA biosynthesis in *Arabidopsis* roots. (*A* and *B*) nlsGPS1 emission ratios of roots 4 d post sowing in WT Col0 or GA biosynthetic mutants *ga3ox1*, *ga3ox2* and *ga20ox1*, *ga20ox2*, *ga20ox3*. (*A*) Representative three-dimensional (3D) ratio and YFP (*Inset*) fluorescence images (scale bar, 30 µm). (*B*) Emission ratios as a function of distance from root tip. Curves of best fit and 95% CIs are computed in R using local polynomial regression (Loess) via ggplot, with smoothing parameter span = 0.75. Complete experiments were repeated at least three times with similar results (*n* = 10 to 30 roots). (*C*) Schematic of the multicellular mathematical model. The model simulates the GA and nlsGPS1 dynamics in a single cell file that represents cell files within the growth zones of the *Arabidopsis* root tip. We prescribe the cells’ growth and division dynamics (*SI Appendix*, Fig. S2): cells divide within the meristem and elongate slowly due to cytoplasmic expansion before ceasing division on entering the elongation zone, where they undergo rapid elongation because of vacuolar expansion. We simulated a system of ordinary differential equations for the cytoplasmic GA concentration in each cell, i, (denoted by [GA]_i_ (t), in terms of time (t)). As shown, the GA dynamics depend on the GA synthesis rate, (σ[x], in which x denotes the distance from the QC), the GA degradation rate, β, the relative elongation rate in the meristem, RER_m_, the relative elongation rate in the elongation zone, RER_EZ_, the proportion of the meristem cells that is cytoplasm, γ, the ratio between vacuolar and cytoplasmic GA concentrations, κ, the cell length, l_i_(t), and the length of the cell on leaving the meristem (L_mi_). We consider GA synthesis rates of the form σ(x)=σ_QC_+αx^n^/(ξ^n^+x^n^), as shown in *D* for *n* = 10, σ_QC_ = 0.00005 and ξ = 125. (*D*) GA synthesis rate distribution for different values of α. EZ, elongation zone. (*E*) Prediction of GA distribution with different α values (corresponding to the synthesis rate distributions shown in *D*). (*F*) Model prediction with a high GA biosynthesis rate in the elongation zone (α = 0.0006) reproduces the distribution observed in the nlsGPS1 data. Model parameter values are given in *SI Appendix*, Table S1. (*G*) Model predictions of nlsGPS1 emission ratios in WT Col0 versus reduced degradation mutant, *ga2ox q*, and reduced synthesis mutant, *ga20ox1*, *ga20ox2*, *ga20ox3*.

As described in the introduction, the predicted GA distribution from our original mathematical model (12) does not agree with our later observations using the nlsGPS1 biosensor (13). Although the model assumed that GA synthesis does not occur in the elongation zone, the increased GA levels observed in these cells led us to hypothesize that substantial GA synthesis does occur there. As we previously reported, roots of GA biosynthesis mutants exhibit shorter meristems, faster initial cell elongation, and reduced length of elongated mature cells (12), but the change in GA distribution in these mutants was not directly measured. We quantified nlsGPS1 emission ratios in *Arabidopsis* roots of two GA biosynthesis deficient backgrounds: the *ga3ox1*, *ga3ox2* double mutant and *ga20ox1*, *ga20ox2*, *ga20ox3* triple mutant. As expected, these highly GA-deficient mutant lines exhibited greatly reduced nlsGPS1 emission ratios in the elongation zone, resulting in a less-pronounced GA gradient from the meristematic zone to the elongation zone (Fig. 1 *A* and *B* and *SI Appendix*, Fig. S1).

To quantitatively investigate our hypothesis that the observed nlsGPS1 emission ratio distribution reflects substantial GA synthesis in the elongation zone, we created a multicellular mathematical model to simulate GA dynamics within both the meristematic and elongation zones. The mathematical model represents the growing region of an *Arabidopsis* root tip as a specified region of dividing cells (the meristem) and an adjacent region of elongating cells (the elongation zone) (Fig. 1*C*). Cells in the meristem elongate slowly and divide at regular time intervals; once a cell leaves the meristematic zone and enters the elongation zone, it ceases division and exhibits rapid cell elongation as it traverses the elongation zone, before ceasing elongation on entering the mature zone. We parameterized these cellular growth dynamics in our conditions by collecting data on root elongation rates and the spatial profile of cell lengths and calculating key growth parameters using the kinematic approach described, for example, in refs. 27 and 28 (*SI Appendix*, Fig. S2 and Table S1 and *Supplementary Information Text*). Within the population of dividing and elongating cells, we represented the GA dynamics via an ordinary differential equation (ODE) within each cell containing terms representing GA synthesis, degradation, and dilution (Fig. 1*C*). We accounted for the subcellular structure by explicitly modeling cytoplasm, nucleus, and vacuole compartments, supposing that cell elongation occurs due to expansion of the cytoplasm and nucleus in the meristem and expansion of the vacuole in the elongation zone. We specified the GA concentrations to be lower in the vacuole based on the pK and pH values (*SI Appendix*, *Supplementary Information Text* and Table S1) (12, 23, 29). Having predicted the GA distribution, we calculated the corresponding nlsGPS1 distribution using the nonlinear relationship suggested from titration curves in Rizza et al. (13) (*SI Appendix*, Fig. S1*B*).

As expected from our previous model (12), with GA biosynthesis in the meristem only, the predicted GA levels are constant in the meristem and reduce as cells traverse the elongation zone (Fig. 1*E*), which is not consistent with the nlsGPS1 data (Fig. 1*B*, black line). We hypothesized that introducing GA synthesis in the elongation zone into our model may counteract the effect of dilution. Specifying the GA synthesis rate to increase as cells traverse the growth zones (using a Hill function to represent a synthesis rate distribution that increases smoothly from a low value at the quiescent center [QC] to a higher value within the mature zone, Fig. 1*D*), the model indeed predicted an increasing GA gradient (Fig. 1*E*).

Quantitatively comparing our model predictions with the biosensor data required us to carefully specify the model parameters. Parameters related to the growth dynamics were calculated from our own growth data (*SI Appendix*, Fig. S2 and Table S1), and parameters related to the pH and dissociation constant were available in the literature (23, 29) (*SI Appendix*, Table S1). However, the minimum and maximum GA synthesis rates ($\sigma_{QC},\alpha$), the position and steepness of the spatial switch between them (ξ,n), and the value of the GA degradation rate (β) are unknown, as these will depend on the in vivo activity levels of the metabolic enzymes. For several values of the degradation rate β and the steepness parameter  n, we found the remaining parameters of GA synthesis ($\sigma_{QC},\alpha ,\xi$) that minimized the error between model predictions and the experimental biosensor data (*SI Appendix*, *Supplementary Information Text* and Table S2). In each case, the nlsGPS1 model predictions and experimental data are in good quantitative agreement (Fig. 1*F* and *SI Appendix*, Fig. S4), but we found the smallest error when the synthesis has a steeper increase between a low value in the basal meristem and a high value in the elongation zone (Fig. 1*D*). Importantly, in all cases the theoretical GA biosynthesis rate in the elongation zone is substantially greater than that in the meristematic zone.

Because *AtGA20ox1* is preferentially expressed in the meristem (12), our model predictions motivated us to investigate the distributions of other biosynthetic enzymes that may explain the longitudinal root GA gradient. We analyzed root cell type– and zone-specific transcriptomic data from Li et al. (25) to determine GA biosynthetic enzymes that show expression patterns that better match, compared to *AtGA20ox1*, nlsGPS1 emission ratios in the elongation zone and the biosynthetic parameters of our multicellular mathematical model (*SI Appendix*, Fig. S3*A*). We also investigated expression patterns of GA biosynthetic enzymes using promoter-GUS fusions from Mitchum et al. and Plackett et al. (16, 26) (*SI Appendix*, Fig. S3 *C* and *D*). In both datasets, and as previously reported, *AtGA20ox1* is expressed in the meristematic zone and not the elongation zone (*SI Appendix*, Fig. S3 *B* and *C*). Interestingly, in both datasets *AtGA3ox2* is expressed in the elongation zone and not the meristematic zone, perhaps indicating this isozyme strongly influences GA distribution in root tips. In the transcriptomic dataset, *AtGA20ox2*, *AtGA20ox3*, and *AtGA3ox1* are expressed in both meristematic and elongation zones, though this was not recapitulated in the promoter-GUS fusions tested, where GUS reporter expression was not detected (*SI Appendix*, Fig. S3 *A* and *B*).

To further refine our model parameter estimates and investigate the role of synthesis and degradation enzymes, we visualized nlsGPS1 in *ga2ox1*, *ga2ox2*, *ga2ox3*, *ga2ox4*, *ga2ox6* quintuple mutant roots, which have reduced GA degradation (*ga2ox q*, lacking five of the GA2ox enzymes involved in GA_4_ catabolism). We observed no substantial differences in endogenous GA distribution compared to wild type (WT) (*SI Appendix*, Fig. S1*C*). Predicting the GA and nlsGPS1 distributions in a *ga2oxq* mutant (assuming this reduces degradation to 10% of the WT value), we found that the model could only reproduce the observed similar GA levels in *ga2oxq* and WT provided the GA degradation rate is small (*SI Appendix*, Fig. S4). Thus, the *ga2oxq* data suggests the parameter set with smaller degradation rate best reflects the GA dynamics *in planta*. We also simulated the GA dynamics in the *ga20ox1*, *ga20ox2*, *ga20ox3* mutant (reduced synthesis in Fig. 1*G* and *SI Appendix*, Fig. S4 *D*, *I*, *N*, and *S*). The model predicted reduced GA levels in the elongation zone, in agreement with the nlsGPS1 data (Fig. 1 *B* and *G*).

### Mapping Gibberellin Biosynthetic Activities Suggests that GA3ox Limits GA_4_ Levels in the Elongation Zone of *Arabidopsis* Roots.

**Fig. 2.**
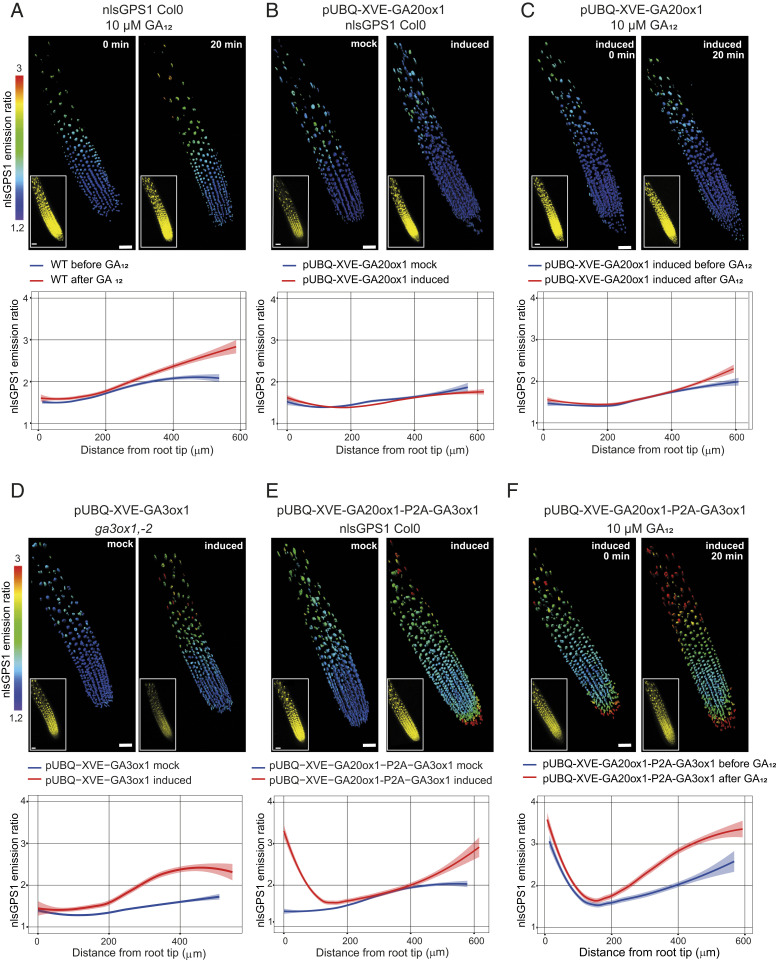
Mapping GA biosynthetic enzyme activities in *Arabidopsis* roots. (*A*–*F*) nlsGPS1 emission ratios of roots 5 d post sowing. Shown are representative 3D images of emission ratios and YFP fluorescence (*Inset*) and the emission ratios as a function of distance from root tip (scale bar, 30 µm). Curves of best fit and 95% CIs are computed in R using local polynomial regression (Loess) via ggplot, with smoothing parameter span = 0.75. Complete experiments were repeated at least three times with similar results *(n* = 15 to 36 roots). (*A*, *C*, and *F*) Before (0 min) and 20 min after 10 µM GA_12_. (*B*–*F*) β-estradiol inducible GA enzyme transgenic lines 24 h after induction with 5 µM 17-β-estradiol (induced) or with 0.2% DMSO mock induction (mock).

To further investigate the model prediction that GA biosynthesis is higher in the elongation zone and that this leads to the observed GA gradient, we mapped whether and where specific steps of GA biosynthesis are limiting, and therefore, contribute to formation of the gradient. We first analyzed early steps in GA biosynthesis by providing roots with high levels of the biosynthetic intermediate GA_12_. When WT *Arabidopsis* roots expressing nlsGPS1, which reports on bioactive GA_4_ but not GA_12_ (13), were treated with 10 µM exogenous GA_12_ for 20 min, a modest increase in bioactive GA was detected only in the elongation zone (Fig. 2*A* and *SI Appendix*, Fig. S6*D*). This result suggested that early biosynthetic steps leading to GA_12_ are not limiting for GA accumulation in the meristematic zone but are limiting in the elongation zone. While expression of most early GA biosynthetic enzymes extends into the elongation zone, the first committed step, ent-copalyl diphosphate synthase (CPS), exhibits high expression in the QC and low expression in the meristematic and elongation zones (*SI Appendix*, Fig. S3). A lack of CPS activity, and thus local GA_12_ synthesis, could contribute to this key intermediate being limiting in the elongation zone. Because GA_12_ is known to be mobile across *Arabidopsis* organs (30), an intriguing possibility is that GA biosynthesis in the elongation zone is dependent on GA_12_ mobilized from the shoot or other root tissues.

We next analyzed the GA20ox step in GA biosynthesis using *Arabidopsis* lines expressing the nlsGPS1 biosensor along with a β-estradiol inducible AtGA20ox1 enzyme (i.e., UBQ:XVE:AtGA20ox1, *SI Appendix*, Fig. S6*A*) (31). The effect of β-estradiol induction of ubiquitous *AtGA20ox1* expression on the endogenous longitudinal GA gradient was minor (Fig. 2*B* and *SI Appendix*, Fig. S6*E*), though GA20ox activity had previously been suggested to be rate-limiting (26). GA_12_ treatment during induction of *AtGA20ox1* expression slightly increased GA in the elongation zone, similarly to treatment with GA_12_ alone (Fig. 2*C* and *SI Appendix*, Fig. S6*F*). Together, these results suggest that GA20ox is not alone limiting GA accumulation in *Arabidopsis* roots.

To analyze the GA3ox step, we evaluated *ga3ox1*, *ga3ox2* mutant lines expressing the nlsGPS1 biosensor along with a β-estradiol inducible AtGA3ox1 enzyme (i.e., UBQ:XVE:AtGA3ox1) (*SI Appendix*, Fig. S6*C*). The effect of β-estradiol induction of ubiquitous *AtGA3ox1* expression on the endogenous longitudinal GA gradient was a substantial GA increase in the elongation zone (Fig. 2*D* and *SI Appendix*, Fig. S6*G*), suggesting that GA3ox activity is limiting in the elongation zone. Taken together, these results indicate that GA3ox activity, along with a contribution from early steps leading to GA_12_, is the primary driver of elongation zone GA levels. In order to confirm this finding, we also compared nlsGPS1 emission ratios in WT roots and roots with either weak or strong overexpression of the AtGA3ox1 enzyme (i.e., mock versus β-estradiol induced UBQ:XVE:AtGA3ox1) (*SI Appendix*, Fig. S7). Ubiquitous overexpression of *AtGA3ox1* alone moderately increased GA levels in the elongation zone, and *AtGA3ox1* in concert with GA_12_ strongly increased GA there (*SI Appendix*, Fig. S7 *B*–*E*). These findings provide further support for the conclusion that *GA3ox* expression and GA_12_ are together limiting in the root elongation zone.

The lack of strong GA increases in the meristematic region during the above perturbations to GA biosynthesis suggests that multiple enzymatic steps are together limiting. Indeed, the effect of simultaneous β-estradiol induction of ubiquitous *AtGA20ox1* and *AtGA3ox1* expression, as compared with the induction of *AtGA3ox1* alone, was a substantial GA increase confined to the rootward meristematic zone near the QC (Fig. 2*E* and *SI Appendix*, Fig. S6*H*). This suggests that GA20ox and GA3ox activity are together limiting in this root region. Interestingly, the QC exhibits expression of all early biosynthetic genes including the strongest expression of *CPS*, perhaps explaining why GA_12_ is not limiting near the QC (*SI Appendix*, Fig. S3).

In contrast, the central meristematic zone did not show strong GA increases during the simultaneous induction of AtGA20ox1 and AtGA3ox1 expression, even with GA_12_ treatment (Fig. 2*F* and *SI Appendix*, Fig. S6*I*). This surprising result suggests further hypotheses regarding regulation of GA biosynthesis in the central meristematic zone: 1) exogenous GA_12_, though highly membrane permeable (23) and provided in excess, is not accumulated, possibly owing to direct GA_12_ catabolism or locally low permeability, or 2) either or both GA20ox and GA3ox are posttranscriptionally downregulated. A third hypothesis, that high GA_4_ depletion activities in these cells limit bioactive GA accumulation to below the detection limit of the biosensor, is not supported by results indicating that this region can accumulate high levels of exogenous GA_4_ (see below).

### An Exogenous GA–Generated Gradient.

**Fig. 3.**
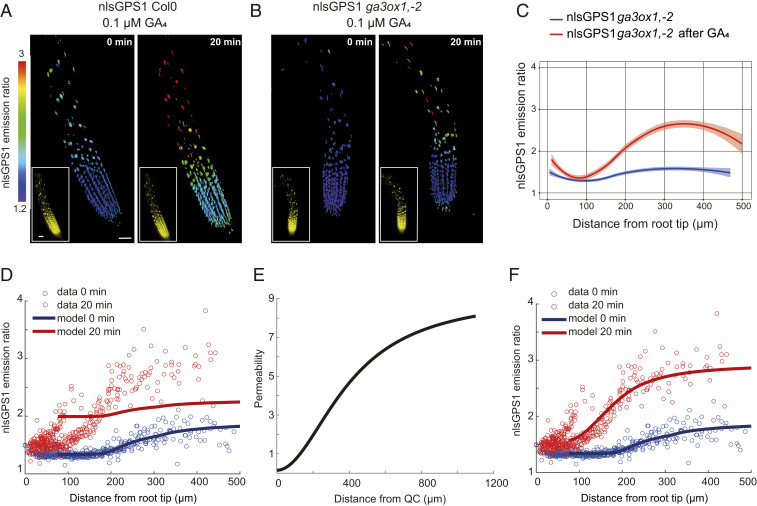
Patterned cellular permeability explains the exogenous GA–generated gradient in *Arabidopsis* roots. (*A* and *B*) Representative 3D images of nlsGPS1 emission ratios and YFP fluorescence (*Inset*) of WT Col0 and *ga3ox1*, *ga3ox2* roots 4 d post sowing before and 20 min after 0.1 µM GA_4_ (scale bar, 30 µm). (*C*) nlsGPS1 emission ratios for nuclei of *ga3ox1*, *ga3ox2* double mutant as a function of distance from the root tip before and after GA_4_. Curves of best fit and 95% CIs are computed in R using local polynomial regression (Loess) via ggplot, with smoothing parameter span = 0.75. Complete experiments were repeated at least three times with similar result (*n* = 10 roots). (*D*) Model prediction and experimental data for the nlsGPS1 distribution after a 0.1 µM GA_4_ dose assuming spatially homogeneous permeability. (*E*) Spatially varying permeability specified in model predictions shown in *F*. (*F*) Model prediction and experimental data for the nlsGPS1 distribution after a 0.1 µM GA_4_ dose, assuming a spatially varying permeability as shown in *E*. Model parameters for *D* and *F* are given in *SI Appendix*, Table S1.

We previously showed that in *Arabidopsis* roots exogenous GA_4_ is also distributed as a gradient, with a faster accumulation of GA_4_ in the elongation zone compared to the meristematic zone (13) (Fig. 3*A*). Therefore, we aimed to investigate the factors and mechanisms controlling the observed exogenous GA distribution. We first tested whether local endogenous GA biosynthesis might play a role in the exogenous GA–generated gradient. Hence, we analyzed the nlsGPS1 emission ratios in roots of *ga3ox1*, *ga3ox2* double mutant after GA_4_ treatment for 20 min (Fig. 3 *B* and *C*). A strong accumulation of GA_4_ was detected in the elongation zone, restoring the longitudinal GA_4_ gradient in this mutant (Fig. 3 *B* and *C*). This result confirmed that the exogenous GA–generated gradient is independent of GA biosynthesis.

We next used our multicellular mathematical model to simulate an exogenous GA treatment by introducing a spatially homogeneous source of GA into the ODEs within each cell. With an exogenous GA treatment that slightly increases GA levels within all cells, the model predicted less GA accumulation than observed in the elongation zone (Fig. 3*D*). Thus, although our model with increased biosynthesis in the elongation zone represented endogenous GA levels well, it did not explain GA levels in response to exogenous GA treatment.

We hypothesized that the gradient generated by exogenous GA could be explained if either 1) the root meristematic zone has higher GA catabolic activity or 2) if the elongation zone has higher cellular permeability to GA. Expression of *GA2ox* catabolic enzymes is low in the meristematic zone, while expression of GA importer *NPF3* is highest in the elongation zone, lending support to the second hypothesis (*SI Appendix*, Fig. S3). To investigate these hypotheses further, we simulated GA treatment of WT Col0 and a mutant with reduced catabolism with either uniform or gradually increasing permeabilities (*SI Appendix*, Fig. S8 *A* and *B*). The uniform permeability simulation predicted that at 20 min treatment GA accumulates at only slightly lower levels in the meristematic zone compared to the elongation zone (*SI Appendix*, Fig. S8*A*). On the other hand, a simulation with gradually increasing permeability can give the observed exogenous GA–generated gradient in WT (*SI Appendix*, Fig. S8*B*). In both simulations, reduced catabolism had minimal effect on the exogenous GA–generated gradient compared to WT (*SI Appendix*, Fig. S8 *A* and *B*).

Testing the model prediction regarding catabolism, we applied GA to the roots of the *ga2ox q* mutant and observed endogenous- and exogenous GA–generated distributions similar to those observed for WT Col0 (*SI Appendix*, Figs. S3 and S8 *C* and *D*). Comparing the model predictions with biosensor data, we found that a gradual increase in permeability between a low value at the QC and a high value in the late elongation zone (Fig. 3*E*) led to excellent agreement between the predictions and data (Fig. 3*F*). Together, these results point to differential permeability as being important for the exogenous GA–generated gradient within the root.

### Cellular Permeability and GA Import Mechanisms.

**Fig. 4.**
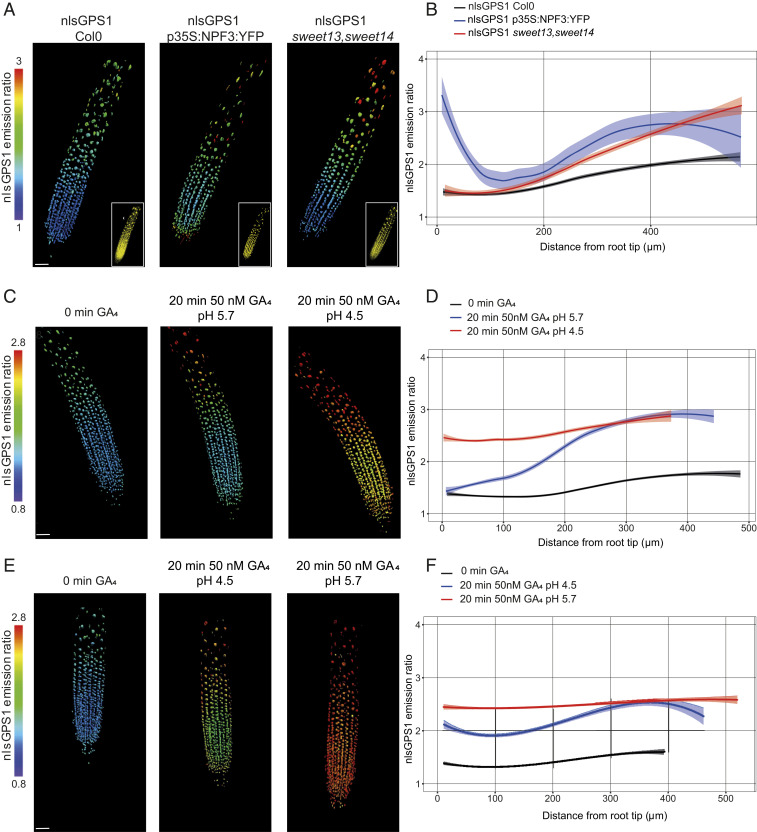
Endogenous GA levels in transporter mutants and exogenous GA accumulation under low pH. (*A*, *C*, and *E*) Representative 3D images of nlsGPS1 emission ratios and YFP fluorescence (*A* only, *Inset*). (Scale bar, 30 µm.) (*B*, *D*, and *F*) Emission ratios as a function of distance from root tip. Curves of best fit and 95% CIs are computed in R using local polynomial regression (Loess) via ggplot, with smoothing parameter span = 0.75. Complete experiments were repeated at least three times with similar results *(n* = 15 to 36 roots). (*A* and *B*) nlsGPS1 emission ratios of roots 4 d post sowing in WT Col0, p35S:NPF3:YFP, and *sweet13, sweet14* double mutant. (*C*–*F*) nlsGPS1 emission ratios of seedlings grown in the RootChip8S. (*C*) Still frames from Movie S1 at 0, 20 min post-GA at pH 5.7, and 20 min post-GA at low pH 4.5. (*E*) Still frames from Movie S2 at 0, 20 min post-GA at low pH 4.5, and 20 min post-GA at pH 5.7.

Permeability of cells to GA is thought to depend in part on transmembrane transporters that are driven by a proton gradient. So far, several members of the NPF family (19, 22) and two SWEET proteins (AtSWEET13 and AtSWEET14) (21) have been shown to function as energy-driven GA importers localized in the plasma membrane. Therefore, we analyzed the nlsGPS1 emission ratios in a 35S:NPF3:YFP transgenic line and a *sweet13*, *sweet14* double mutant (Fig. 4 *A* and *B* and *SI Appendix*, Fig. S9). Overexpression of *NPF3* led to broadly higher GA levels, with particularly elevated GA in the rootward meristematic and elongation zones (Fig. 4 *A* and *B*). Surprisingly, loss of SWEET13 and SWEET14 also led to higher GA levels, though only in the elongation zone (Fig. 4 *A* and *B*). One possible explanation for this result is that loss of endogenous SWEET-driven GA import in inner cell types—SWEET13 and SWEET14 are expressed strongly in the stele (21)—results in increased GA_4_ pools in the apoplasm of the epidermal and cortical cells, where our nlsGPS1 biosensor measurements are focused. However, because both transporter types also transport other substrates (e.g., nitrate and other plant hormones for NPFs and sugars for SWEETs), and because both mutants have developmental phenotypes, we must also consider the possibility that the effects we observe on GA levels are the result of perturbations in these other substrates indirectly affecting other GA biochemical steps or transport mechanisms (e.g., acid-trap).

Because all known mechanisms of cellular permeability to GA rely on low extracellular pH relative to the cytosol, we hypothesized that if the exogenous GA–generated gradient was driven by permeability patterns, then manipulating extracellular pH would perturb the gradient. Thus, we analyzed the nlsGPS1 emission ratio in roots growing in a modified RootChip-8S microfluidic device in which extracellular pH and GA levels can be rapidly manipulated (32). We examined the exogenous GA–generated distribution at standard pH (pH 5.7) followed by low pH (pH 4.5). As observed previously, at pH 5.7, we observed exogenous GA_4_ accumulation in all zones, with faster accumulation in the elongation zone (Fig. 4 *C* and *D* and Movie S1). At low pH, we observed a strong and fast accumulation of exogenous GA_4_ in the meristematic zone (Fig. 4 *C* and *D* and Movie S1), suggesting that raised apoplasmic pH in the meristematic zone limits exogenous, and potentially also endogenous, GA_4_ accumulation. We confirmed the strong and fast accumulation of GA_4_ in the meristematic zone at low pH by performing the reverse experiment with exogenous GA_4_ at pH 4.5 followed by pH 5.7 (Fig. 4 *E* and *F* and Movie S2). We did not observe any substantial differences in the endogenous GA distribution at standard pH versus low pH (Movie S3), suggesting that low pH alone does not change GA levels or otherwise affect biosensor emission ratios under the timescale and conditions tested. Taken together, our theoretical and experimental results suggest that differential cell permeability is key for establishing the exogenous GA–generated gradient. A pH driven longitudinal differential in cellular permeability for GA is in agreement with previous works that showed a longitudinal differential in apoplast acidification correlated with cell elongation in roots (33, 34).

### GA Gradient and Cellular Growth.

**Fig. 5.**
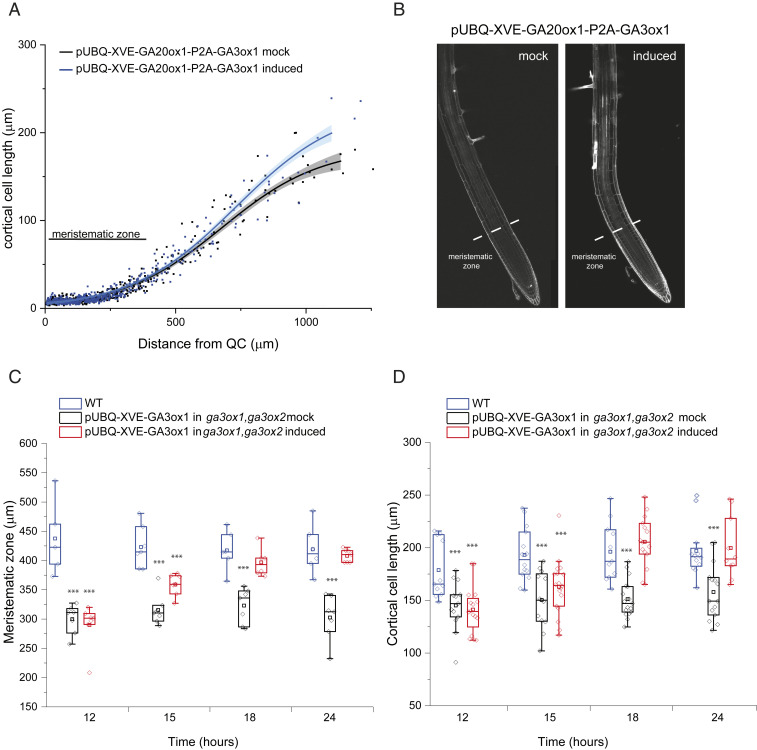
GA root growth phenotypes with GA overproduction and complementation. (*A* and *B*) Five-day-old roots of nlsGPS1 in pUBQ-XVE-AtGA20ox1-P2A-GA3ox1 line after 24 h with mock (0.2% DMSO) or 5 µM 17-β-estradiol induction. (*A*) Cortical cell length from the QC to the elongation zone with the border between meristematic zone and elongation zone indicated. Curves of best fit and 95% CIs are computed in R using local polynomial regression (Loess) via ggplot, with smoothing parameter span = 0.75. (*B*) Representative images of PI-stained roots with the border between meristematic zone and elongation zone indicated. (*C* and *D*) Beeswarm and box plots of growth phenotypes for 5-d-old roots of nlsGPS1 in either WT or a pUBQ-XVE-AtGA3ox1 in *ga3ox1, ga3ox2* mutant line after 12, 15, 18 and 24 h induction with 5 µM 17-β-estradiol or mock solution (0.2% DMSO). Asterisks indicate a significant difference between treatments and untreated WT (one-way ANOVA test; ***P < 0.001). (*C*) Meristematic zone length including root cap. (*D*) Mature cortical cell length (950 µm from the QC).

As previously mentioned, deficiency in GA biosynthesis caused gross phenotypes in the architecture of *Arabidopsis* roots, with shorter meristem size and reduced length of elongated mature cells (11). Using these phenotypes, we investigated the spatial and temporal relationship between establishment of GA gradients and cellular growth. Interestingly, cell division and elongation patterns in the meristem and early elongation zone are not affected by the simultaneous induction of *AtGA20ox1* and *AtGA3ox1* expression (Fig. 5 *A* and *B*), despite elevated GA levels in the rootward meristematic zone and an increased slope of the GA gradient in elongation zone (Fig. 2*E*). Thus, the correlation between GA levels and cellular growth in roots might not represent a simple dose–response mode of GA action in which locally increased GA concentrations drive increased cell elongation. For example, if the cellular GA gradient is sufficient to saturate GA signaling in WT roots, then additional GA would not greatly affect root growth. The absence of a simple GA dose–response relationship in root growth has previously been observed in several plant species (35).

Nonetheless, it is striking that endogenous-GA gradients are maintained even with the overexpression of GA biosynthetic enzymes and correlate well with exogenous GA–generated gradients, despite deriving from distinct mechanisms, perhaps suggesting a functional consequence for GA depletion from, and consequential DELLA accumulation within, the meristematic zone. In order to examine the functional consequences of DELLA accumulation in the *Arabidopsis* meristematic zone, we examined root meristem length in a quintuple *della* mutant (*global*) compared to WT (Ler). Interestingly, there was no substantial difference (*SI Appendix*, Fig. S10 *A* and *B*), suggesting that DELLA accumulation and GA depletion in the meristematic zone may not be required for root tip cell division and elongation patterns in *Arabidopsis* under the conditions tested. Though we cannot exclude the possibility of residual *della* activity in the *global* mutant, this result is also consistent with GA levels being sufficient to saturate GA signaling in WT roots.

Thus, we propose a cooperative local dose–response relationship in which GA concentration in elongating cells acts in concert with other signals (i.e., is necessary but not sufficient) to direct cell elongation. However, we also demonstrate that complementation of GA deficiency phenotypes is substantially delayed compared with complementation of GA levels. We analyzed meristem length (Fig. 5*C* and *SI Appendix*, Fig. S11) and elongated cortical cell length (Fig. 5*D* and *SI Appendix*, Fig. S12) of roots after 12, 15, 18, and 24 h of mock or β-estradiol induction of *AtGA3ox1* expression in a *ga3ox1*, *ga3ox2* double mutant. A total of 4 h of β-estradiol treatment is sufficient to fully rescue the GA gradient (*SI Appendix*, Fig. S12), and it takes approximately 4 h for a cell to traverse the elongation zone (12), but long-term treatment was necessary for rescuing the root meristem and cell length phenotypes (Fig. 5 *C* and *D*). Over 15 h of *AtGA3ox1* induction was necessary to fully rescue the size of the meristematic zone and elongated cells (Fig. 5 *C* and *D*). The substantial temporal delay between forming a GA gradient and GA dependent growth responses suggests that, rather than a cooperative local dose–response relationship, a hysteresis model might better represent the quantitative relationship between GA levels and growth. Specifically, GA might influence cell elongation via prior signaling in meristematic zone cells.

### Concluding Remarks.

The longitudinal GA gradients, endogenous- and exogenous GA–generated, in *Arabidopsis* roots can be considered as the combined effect of GA metabolism, transport, and dilution. Here, we present and test a multicellular mathematical model to understand how such processes combine to produce the observed GA distributions. The model revealed that the endogenous-GA gradient reflects GA synthesis increasing as cells traverse the growth zones, whereas the exogenous-GA-generated gradient reflects cellular permeability to GA increasing as cells traverse the growth zones. Comparing model predictions to biosensor data for different parameter sets led to quantitative predictions for the distribution of synthesis rates and the cellular permeability to GA, revealing both to increase over 10-fold across the growth zones. Furthermore, the model presented provides a framework for future simulations of hormone dynamics, which are often tightly regulated by myriad biochemical steps, within organs with distinct growth zones.

Our study also provides evidence that GA accumulation in the rootward meristematic zone is limited by the expression of *GA3ox* and *GA20ox* biosynthetic enzymes, while in the elongation zone GA is limited by *GA3ox* expression combined with levels of the GA_12_ precursor. At present, we can only speculate at the mechanism limiting GA biosynthesis in the central meristematic zone, as excess precursor- and rate-limiting enzyme overexpression are not sufficient to drive bioactive GA overaccumulation. One possibility is that the GA20ox and GA3ox enzymes require 2-oxoglutarate as cosubstrate and this master regulator primary metabolite, which has been found to affect GA levels (36–38), could be limited in meristematic tissues. Together, these findings suggest several mechanistic avenues for environmental cues to influence spatial GA distribution in roots, with *GA3ox* expression and GA_12_ translocation from other tissues being a likely target for elevating GA levels, though GA depletion from sites of accumulation is perhaps more likely to yield consequential phenotypes for *Arabidopsis*. Time course analyses of GA and root growth complementation in a GA biosynthetic mutant revealed that the influence of GA on cell expansion in roots is sufficiently slow that we cannot at present discriminate between a cooperative local dose–response model or a distal hysteresis model of GA control over root cell elongation. Nonetheless, the absence of GA from certain root zones is likely unimportant for patterning cell division and growth, at least in rapidly growing *Arabidopsis* roots under the conditions tested. It remains possible that GA would direct cell elongation in a more simple concentration-dependent manner in other tissues or species (e.g., *Arabidopsis* hypocotyls or rice stems in which higher GA levels increase elongation).

## Materials and Methods

### Plant Material and Growth Conditions.

WT, mutant, and transgenic lines used in this study were *A. thaliana* ecotype Columbia 0 (Col-0) except for the *global* mutant and control, which is Landsberg erecta (Ler-0). Seeds were chlorine-gas-sterilized and plated on 1/2 Murashige and Skoog (MS) basal medium (Duchefa, Cat No. M0221) with 0.025% 2-morpholinoethanesulfonic acid monohydrate (MES), pH 5.7, and 0.8% agar (1/2 MS solid medium, Sigma). After stratification in the dark at 4 °C for 2 d, plates were placed in a growth chamber with long-day growth conditions (16 h light/8 h dark cycling, temperature cycling 22 °C day/18 °C night, and 70% relative humidity). For β-estradiol induction of pUBQ-XVE-AtGA20ox1, pUBQ-XVE-AtGA20ox1-P2A-AtGA3ox1, and pUBQ-XVE-AtGA3ox1 lines, 4-d-old seedlings were transferred to 1/2 MS solid medium supplemented with 5 µM 17-β-estradiol for 24 h prior to confocal imaging. Mock induction with dimethyl sulfoxide (DMSO) was used as control. The induction of GA enzyme expression was confirmed by qRT-PCR (*SI Appendix*, Fig. S6 *A*–*C*). The following Arabidopsis lines generated in this work have been deposited with the Nottingham Arabidopsis Stock Centre with the following NASC IDs. N2110211: *ga20ox1*,*ga20ox2*,*ga20ox3* nlsGPS1; N2110212: *ga2ox* *q* nlsGPS1; N2110213: pUBQ10-XVE-GA20ox1 nlsGPS1; N2110214: pUBQ10-XVE-GA3ox1 nlsGPS1; N2110215: pUBQ10-XVE-GA3ox1 nlsGPS1 in *ga3ox1*, *ga3ox2*; N2110216: pUBQ10-XVE-GA20ox1-P2A-GA3ox1 nlsGPS1; N2110217: *sweet13,14* nlsGPS1; N2110218: 35S:NPF3:YFP nlsGPS1.

### Cloning Strategy and Generation of *Arabidopsis* Transgenic Lines.

Coding sequences of *AtGA20ox1* and *AtGA3ox1*, were amplified by using primers listed in *SI Appendix*, Table S3 and the resulting PCR products were recombined into pDONR221 in a BP reaction. For the coexpression of both genes (*AtGA20ox1*, *AtGA3ox1*), the ribosome skipping sequence (P2A, porcine teschovirus-1 2A) was incorporated between the two genes by overlap extension PCR. In the first step, *AtGA20ox1* sequences were amplified with 5′ primers containing the attB1 site and 3′ primers containing an 18-base-pair sequence for overlapping PCR. *AtGA3ox1* sequences were amplified with 5′ primers containing the same 18-base-pair sequence for overlapping PCR and 3′ primers containing the attB2 site. In the second step, two products from the first reactions were amplified together in an overlap extension PCR and the resulting product was recombined into pDONR221 in a BP reaction. The resulting pEntry clones for the corresponding GA biosynthetic genes, the AtUBQ10 promoter, and the terminator sequences, were recombined into promoter-less p1R4-ML:XVE binary expression vector (31) using a MultiSite Gateway reaction. Transgenic plant lines (pUBQ-XVE-AtGA20ox1, pUBQ-XVE-AtGA20ox1-P2A-AtGA3ox1, and pUBQ-XVE-AtGA3ox1) were generated using the Agrobacterium floral dip and transformants were selected on agar plates containing 1/2 × MS medium with Hygromycin.

### RNA Extraction and Expression Analysis.

For β-estradiol induction of pUBQ-XVE-AtGA20ox1, pUBQ-XVE-AtGA20ox1-P2A-AtGA3ox1, and pUBQ-XVE-AtGA3ox1 lines, RNA extraction was performed using RNeasy Plant Mini Kit (QIAGEN). After DNase digest (Thermo Fisher Scientific), 1 mg total RNA was used for cDNA synthesis with oligo (dT) primers (Transcriptor First Strand cDNA Synthesis Kit, Roche). The expression of T3 homozygous lines was tested by qRT-PCR and the primers used are listed in *SI Appendix*, Table S3.

### Histochemical GUS Staining.

Five-day-old seedlings were incubated for 4 h at 37 °C in the GUS buffer (200 mM NaH_2_PO4, 200 mM Na_2_HPO4, pH 7, 0.5 M EDTA pH 8, 0.1% Triton-X, 20 mM Potassium ferricyanide, and 20 mM Potassium ferrocyanide) containing 20 mM 5-bromo-4-chloro-3-indolyl glucuronide. To stop the GUS reaction, the reaction mix was replaced with an ethanol concentration series (30 min minimum) at 30%, 50%, 70%, and 100%. Seedlings were transferred to a slide with chloral hydrate solution (8 g chloral hydrate, 1 mL glycerol, and 2 mL water) and imaged with an Axio-imager microscope.

### Confocal Imaging.

Sample preparation and mounting was as described in detail previously (13, 39). In brief, for steady-state experiments, seedlings were placed in liquid 1/4 × MS medium (1/4 × MS salts, 0.025% MES, and pH 5.7) with coverslips and imaged. In the experiments with low pH, HCl was added to the liquid media before filter sterilization, as described in detail previously (40). For the GA_4_ (Gibberellin A4 BioReagent, Sigma-Aldrich) and GA_12_ (Gibberellin A12, OlChemIm) treatments, the standard medium beneath the coverslip was exchanged with the medium containing GA solution. Confocal images were acquired with a format of 1024 × 512 pixels and resolution of 12 bit on an upright Leica SP8 using a 20× dry 0.70 HC PLAN APO objective. To excite Cerulean and Aphrodite, 448 nm and 514 nm lasers were used, respectively. The 552 nm laser line was used to excite propidium iodide (PI, Sigma-Aldrich). Emission filters were 460 to 500 nm for Cerulean, 525 to 560 nm for Aphrodite, and 590 to635 nm for PI. Three fluorescence channels were collected for FRET imaging: Cerulean donor excitation and emission or DxDm, Cerulean donor excitation, Aphrodite acceptor emission or DxAm, and Aphrodite acceptor excitation and emission or AxAm. The laser power was set to 3% to excite Cerulean and 2% to excite Aphrodite with detector gain set to 110.

### RootChip18S Devices, Root Perfusion, and Confocal Imaging.

The RootChip18S device was used as described in detail previously (30). Briefly, *Arabidopsis* seedlings were germinated on 5-mm long portion of 10 µl pipette tips filled with solidified growth medium (1/2 × MS salts, 1% Agar, 0.05% (wt/vol) MES, and pH 5.7 supplemented with vitamins). After 4 to 5 d, seedlings were placed onto the polydimethylsiloxane RootChip-8S device under sterile conditions. By using a peristaltic pump (DNE GmbH; volumetric flow rate in each channel, 5 mL/min), the device was perfused with liquid media and the time for treatments to reach the root chamber was measured to be ∼10 min. RootChip imaging was performed by using a 20× dry 0.70 HC PLAN APO objective on an inverted Leica SP8 and 448 nm and 514 nm lasers were used for excitation of Cerulean and Aphrodite, respectively. Emission filters were 460 to 490 nm for Cerulean and 520 to 550 nm for Aphrodite. The laser power was set to 3% to excite Cerulean and 2% to excite Aphrodite with detector gain set to 110, a Z-stack of 2 µm steps, and imaging intervals of 5 min. RootChip imaging was set to 150 min with the following treatment: mock treatment (pH 5.7) for 20 min, 0.05 µM GA_4_ treatment at standard pH (pH 5.7) for 20 min, mock treatment for 30 min, 0.05 µM GA_4_ treatment at pH 4.5 for 20 min, and final mock treatment for 60 min. The same time course was repeated, except for reversing the treatments with exogenous GA_4_ at pH 4.5 followed by pH 5.7. A control time course without exogenous GA was also performed.

### Image Processing and Analysis.

Image processing and analysis were performed by using Imaris 8.3.1 (Bitplane), as described previously (13, 36). The AxAm channel, presented as yellow fluorescent protein (YFP) fluorescence images, was segmented to select for nuclear signals by using the “surfaces wizard” and the following settings: background subtraction (local contrast) set to 3 µm, thresholding set as default. Exceptionally, the YFP fluorescence images in the p35S:NPF3:YFP background were instead generated by segmenting nuclei from the DxAm channel and using this image as a mask for the YFP channel. The ratio channel (DxAm/DxDm) was calculated by using the Imaris Xtension “XT Mean Intensity Ratio.”

### Root Growth Measurements.

Six-day-old roots stained with PI (Fig. 5 and *SI Appendix*, Fig. S2) were analyzed by using ImageJ software. We measured the length of the meristematic zone and cortical cells of the late elongation zone (950 µM from the QC) of 6-d-old roots of WT or pUBQ-XVE-AtGA3ox1 line in *ga3ox1*, *ga3ox2* mutant background. We also measured cortical cells of pUBQ-XVE-AtGA20ox1-P2A-AtGA3ox1 after either mock or 17-β-estradiol induction, starting from the QC until elongation zone. Cortical cells were measured starting from QC of 6-d-old roots of WT, *ga20ox1*, *ga20ox2*, *ga20ox3* and *ga3ox1*, *ga3ox2* mutant backgrounds. Roots were stained with PI. The root growth rate of WT and biosynthesis mutants (*SI Appendix*, Fig. S2) was tracked for 3 d from 5 d post germination until 7 d post germination. Plates were scanned and roots were measured using ImageJ.

### Modeling.

Derivation of the model equations and details of how the model parameter values were estimated are provided in *SI Appendix*, *Supplementary Information Text*. A list of model parameter values can be found in *SI Appendix*, Tables S1 and S2.

## Supplementary Material

Supplementary File

Supplementary File

Supplementary File

Supplementary File

## Data Availability

Biological data and modeling information have been deposited in Apollo Open Access Cambridge Data Repository (https://doi.org/10.17863/CAM.58366) (41).
