# Supplementary material for: Differential biosynthesis and cellular permeability explain longitudinal gibberellin gradients in growing roots
Source: Proc Natl Acad Sci U S A. 2021 Feb 18;118(8):e1921960118. doi: 10.1073/pnas.1921960118 (PMC7923382; doi:10.1073/pnas.1921960118)
Supplement: Supplementary File [file pnas.1921960118.sapp.pdf]

PNAS
www.pnas.org

Supplementary Information for

Differential biosynthesis and cellular permeability explain longitudinal gibberellin gradients in growing roots

Annalisa Rizza[a], Bijun Tang[a], Claire E. Stanley[b,c], Guido Grossmann[d], Markus R. Owen[e], Leah R. Band[e,f,1] and Alexander M. Jones[a, 1]

[a] Sainsbury Laboratory, Cambridge University, Cambridge CB2 1LR, United Kingdom; [b]Institute for Chemical and Bioengineering, 8046 Zürich, Switzerland; [c]Agroecology and Environment Research Division, Agroscope, 8093 Zürich, Switzerland; [d]Centre for Organismal Studies (COS) Heidelberg, 69120 Heidelberg, Germany; [e]Centre for Mathematical Medicine and Biology, School of Mathematical Sciences, University of Nottingham, Nottingham NG7 2RD, United Kingdom [f]Division of Plant and Crop Sciences, University of Nottingham, Sutton Bonington LE12 5RD, United Kingdom;

[1] Corresponding authors: alexander.jones@slcu.cam.ac.uk and leah.band@nottingham.ac.uk

This PDF file includes:

- Supplementary text
- Figures S1 to S12
- Tables S1 to S3
- Legends for Movies S1 to S3
- SI References

Other supplementary materials for this manuscript include the following:

- Movies S1 to S3

Supplementary Information Text
Model description

This document describes the derivation of the cell-based mathematical model that predicts the gibberellin (GA) distribution in the growth zone of an Arabidopsis root.

To create a cell-based model, we exploited the simple architecture of the Arabidopsis root and represented the root's growth zone as a single file of cells, labelled i = 1, 2, ...N . As described below, we simulated the cell-growth dynamics and simulated ODEs that represent GA biosynthesis, degradation and dilution, within this population of cells to predict the spatial GA distribution. The simulations were performed in matlab. The model parameter values and references are provided in Table S1.

1 Simulating the root-tip growth dynamics using a cell-based model

The growth zone of the Arabidopsis root tip comprises three spatially distinct developmental zones: the meristem, the elongation zone and the mature zone. We focused on mature plant roots in which the sizes of the meristem and elongation zone were stable (noting that for Arabidopsis plants, this stabilization occurs approximately 6 days after germination in our growth conditions). Thus, in the frame of reference moving with the start of the elongation zone, growth is quasi-steady and the spatial distributions of cell lengths and growth rates are independent of time. Within the growth zones, cell growth is anisotropic, and thus, we consider cells to have a time-dependent cell length but constant radius. In prescribing the cells' growth dynamics, we followed the results of Van der Weele et al 2003, and take the cells' relative elongation rate (RER) to be constant within each developmental zone. Using the RER values, the cell lengths evolve according to the following standard formula

$$\frac{1}{l_i}\frac{\mathrm{d}l_i}{\mathrm{d}t} = \mathrm{RER}, \tag{1.1}$$

these equations were solved using matlab ode solver *ode*45 during the time intervals between successive division events.

Cells in the meristem elongate slowly and divide at approximately regular time intervals. Based on the data in Beemster and Baskin 1998, cell division is stochastic; the time interval between the time at which

each cell is created the time when it next divides is chosen randomly from a normal distribution with mean T_c hours and standard error T_{cse}. For simplicity, we supposed that the mean cell division rate is constant throughout the meristem. On division, the daughter cells have half the length of the parent cell and a GA concentration equal to the parent cell. We track the positions of the cell centres. Cells move away from the root tip due to elongation of the more rootward cells and the division event only occurs provided the cell centre is within the meristem region.

When a cell leaves the meristem and enters the elongation zone, it ceases division and undergoes rapid elongation (according to formula (1.1)). As detailed in the subsequent section, the ODE representing the GA dynamics in the elongation zone requires us to record the length of each cell i at the time it leaves the meristem, L_{mi}.

Simulations are started with two cells, which grow and divide until a large number of cells are produced and the dynamics have reached a quasisteady state in the frame of reference moving with the root tip. In the results presented, we simulated the model until 150 cells are produced - increasing this number to 300 makes no noticeable difference to the model predictions.

2 Specifying the growth dynamics using data

In order to accurately simulate the growth dynamics in our experimental conditions, we collected data to parameterise the root growth kinematics for Col0, *ga20ox-1,-2,-3* and *ga2ox q*. Analysing growth kinematics in this way has a long history; similar analysis can be found in Beemster and Baskin, 1998 and references therein.

We first fit a simple piecewise linear function to measurements of cell lengths in terms of distance from the QC. Since we are assuming that the RER is constant within the meristem and EZ, and that the division rate is constant in the meristem, it is appropriate to assume that the average cell length is constant in the meristem, increases linearly with distance through the EZ, before becoming constant at the end of the elongation zone (see equations (2.5) and (2.6) below). This piecewise linear function can be characterised by four parameters: the length of the meristem, x_m, the length of the EZ, x_{EZ}, the average cell length in the meristem, L_m, and the mature cell length, L_{mat}. For each case, these four parameters were fitted to the cell-length data using Matlabs inbuilt *fminsearch* algorithm using multiple starting points. The fitted piecewise linear distributions showed reasonable agreement with the cell length measurements in Col0, *ga20ox-1,-2,-3* and *ga2ox q*, see Fig S2D-F.

Using the fitted cell-length distribution together with measurements of the root elongation rate (obtained by measuring root lengths as 5 and 7 days post-germination, see Fig S2C,G), we can calculate T_c the average time interval between successive cell divisions as follows. Since growth is quasi-steady in the frame of reference moving with the root tip, the root elongation rate equals the mature cell length, L_m, divided by the time interval for successive cells entering/leaving the elongation zone, *c*. Thus, we can calculate this

time interval, c, via

$$c = \frac{L_m}{\text{Root elongation rate}}. \tag{2.1}$$

We note that $1/c$ is often called the total cell production rate, i.e. the rate at which new cells are created by the meristem. We can then calculate the average cell division rate, D, by dividing the total cell production rate by the number of meristem cells (given by x_m/L_m):

$$\text{Average cell division rate, } D = \frac{L_m}{cx_m}. \tag{2.2}$$

By the law of exponential growth, the number of cells at time t equals $N_0 \exp(Dt)$ (where N_0 is the number of cells at time 0). Since the cell number doubles during the average cell cycle duration (i.e. time interval between successive cell division events), T_c, we therefore obtain

$$T_c = \frac{\ln(2)}{D}. \tag{2.3}$$

(see Beemster and Baskin, 1998).

Having calculated c and T_c, we can then calculate the RER in each zone as follows. Integrating equation (1.1), provides a formula for the cell length at time t_2, $l(t_2)$, in terms of the cell length at a previous time t_1, $l(t_1)$, and the relative elongation rate in the meristem, RER_m

$$l(t_2) = l(t_1) \exp(\text{RER}_m(t_2 - t_1)); \tag{2.4}$$

using this formula and supposing that during the time interval between successive cell divisions, T_c, a cell has doubled in length (*i.e.* $l(t_2) = 2l(t_1)$) we can calculate RER_m via

$$\text{RER}_m = \frac{\ln(2)}{T_c}. \tag{2.5}$$

In the elongation zone, the RER can be calculated via

$$RER_{EZ} = \frac{1}{l}\frac{dl}{dt} = \frac{1}{c}\frac{dl}{dx}; \tag{2.6}$$

(a derivation of which we previously described in the Supplementary Material of Band et al. 2012). Thus, since we are assuming cell lengths increase linearly within the EZ, the RER can be calculated via

$$RER_{EZ} = \frac{1}{c}\frac{L_{mat} - L_m}{x_{EZ}}. \tag{2.7}$$

Using the above formulae, we obtained the model growth parameters for each case, given in Table S1.

3 Simulating the GA dynamics in the cell-based model of the growing Arabidopsis root tip

Following the method described in the Supplementary material of Band et al 2012, we now derive a system of ordinary differential equations (ODEs) that describe GA synthesis, degradation and dilution accounting for the subcellular structure.

Within each cell, GA is present in both protonated and anionic forms within the vacuole, nucleus and cytoplasm. We assume that within each of these compartments diffusion is sufficiently rapid that spatial variations within a compartment can be neglected, and thus denote the GA concentration in the cytoplasm by $[\mathrm{GA}]_i$, the nucleus by $[\mathrm{GA}]_{nuci}$ and the vacuole by $[\mathrm{GA}]_{vaci}$.

Transport between the nucleus and the cytoplasm is passive, whereas transport between the cytoplasm and the vacuole is controlled by the tonoplast; as is typical in hormone transport models, we assume that the tonoplast is impermeable to anionic GA, but that protonated GA can passively diffuse across it. Thus, the rate at which GA moves between the cytoplasm and vacuole depends on the difference between the cytoplasmic protonated GA concentration, given by $[\mathrm{GA}]_i(1+10^{pH_{cyt}-pK})^{-1}$, and the vacuolar protonated GA concentration, given by $[\mathrm{GA}]_{vaci}(1+10^{pH_{vac}-pK})^{-1}$, where pH_{cyt} and pH_{vac} denote the pH of the cytoplasm and vacuole respectively, and pK denotes GA's dissociation constant.

GA synthesis and degradation are taken to occur in the cytoplasm since enzymes involved in degradation and the later steps of biosynthesis reside here (Olszewski et al, 2002). Thus, the GA concentrations within each subcellular compartment for cell i can be described by

$$\frac{\mathrm{d}(V_{nuci}[\mathrm{GA}]_{nuci})}{\mathrm{d}t} = P_{nuc}([\mathrm{GA}]_i - [\mathrm{GA}]_{nuci}), \tag{3.1a}$$

$$\frac{\mathrm{d}(V_{cyti}[\mathrm{GA}]_i)}{\mathrm{d}t} = P_{nuc}\big([\mathrm{GA}]_{nuci} - [\mathrm{GA}]_i\big) + P_{ton}A_{ton}\left(\frac{[\mathrm{GA}]_{vaci}}{1+10^{pH_{vac}-pK}} - \frac{[\mathrm{GA}]_i}{1+10^{pH_{cyt}-pK}}\right) + V_{cyti}(\sigma - \beta[\mathrm{GA}]_i), \tag{3.1b}$$

$$\frac{\mathrm{d}(V_{vaci}[\mathrm{GA}]_{vaci})}{\mathrm{d}t} = P_{ton}A_{ton}\left(\frac{[\mathrm{GA}]_i}{1+10^{pH_{cyt}-pK}} - \frac{[\mathrm{GA}]_{vaci}}{1+10^{pH_{vac}-pK}}\right), \tag{3.1c}$$

where $V_{nuci}(t)$, $V_{cyti}(t)$ and $V_{vaci}(t)$ are, respectively, the volume of the cell nucleus, cytoplasm and vacuole of cell i at time t, P_{nuc} is the rate at which GA moves between the nucleus and cytoplasm, A_{ton} is the tonoplast area, P_{ton} is the permeability of the tonoplast to protonated GA, σ is the GA synthesis rate and β is the GA degradation rate. To represent the lower synthesis in the meristem and higher synthesis in the elongation zone, we suppose that the GA synthesis rate, σ depends on the distance from the QC, x, as detailed below.

We suppose, for simplicity, that transport across the tonoplast is rapid (i.e. taking $P_{ton}A_{ton}$ to be large), so that the cytoplasmic and vacuole concentrations of protonated gibberellin can be treated as equal (see equation (3.1c)), and therefore, letting κ denote the ratio between the gibberellin concentrations in the vacuole and cytoplasm, we have

$$[\mathrm{GA}]_{vaci} = \kappa[\mathrm{GA}]_i \quad \text{where} \quad \kappa = \frac{1+10^{pH_{vac}-pK}}{1+10^{pH_{cyt}-pK}} \tag{3.2}$$

In addition, we assume that transport between the cytoplasm and nucleus is rapid. Thus, we take P_{nuc} to be large, which suggests that

$$[\mathrm{GA}]_{nuci} = [\mathrm{GA}]_i. \tag{3.3}$$

Under the limits implied by the above assumptions, the model (3.1) then simplifies to equation

$$\frac{d(V_{nuci} + V_{cyti} + \kappa V_{vaci})[\mathrm{GA}]_i}{dt} = V_{cyti}(\sigma - \beta[\mathrm{GA}]_i) \tag{3.4}$$

for the cytoplasmic and nuclear gibberellin concentration (this being the sum of equations 3.1 a-c).

3.1 Meristem equations

Simulating (3.4) required us to specify how the volumes of the subcellular compartments evolve as the cells grows. Within the meristem, the volume of the vacuole is small and we therefore approximate the cells to be comprised of nucleus and cytoplasm compartments. In dividing cells, studies have shown that as the cells elongate, both the nucleus and cytoplasm compartments expand, such that the cytoplasm is a constant proportion of the cell's volume, denoted by γ (Willis et al, 2016; Sugimoto-Shirasu and Roberts, 2003). Thus, we set

$$V_{cyti} = \gamma\pi R^2 l_i, \quad V_{nuci} = (1-\gamma)\pi R^2 l_i, \quad V_{vaci} = 0, \tag{3.5}$$

where R denotes the cell radius (which it is appropriate to take to be constant). Therefore, in the meristem, equation (3.4) reduces to

$$\frac{d[\mathrm{GA}]_i}{dt} = \gamma(\sigma - \beta[\mathrm{GA}]_i) - \mathrm{RER}_m[\mathrm{GA}]_i, \tag{3.6}$$

where we have used equation (1.1). Here the first term of the right-hand-side represents synthesis and degradation, whereas the second term represents dilution as the cytoplasm and nucleus expand.

3.2 Elongation-zone equations

Within the elongation zone, the increase in cell volume is due primarily to an increase in vacuole volume. Thus, as cells elongate, gibberellin within the vacuole dilutes, leading to an influx of protonated gibberellin from the cytoplasm which leads to a reduction in gibberellin concentration within both the cytoplasm and nucleus. We take the simplest assumption and suppose that the cytoplasm and nuclear volumes are constant, and therefore

$$V_{cyti} = \gamma\pi R^2 L_{mi}, \quad V_{nuci} = (1-\gamma)\pi R^2 L_{mi}, \quad V_{vaci} = \pi R^2(l_i - L_{mi}), \tag{3.7}$$

where L_{mi} denotes the length of the cell i when it passes the boundary between the meristem and elongation zone. We note that these elongation-zone assumptions are the same as those in our previous model of the GA dynamics in the elongation zone (Band et al 2012). Thus, in the elongation zone, equation (3.4) reduces to

$$\frac{d[\mathrm{GA}]_i}{dt} = \frac{1}{L_{mi} + \kappa(l_i - L_{mi})}\Big(\gamma L_{mi}(\sigma - \beta[\mathrm{GA}]_i) - \kappa l_i \mathrm{RER}_{EZ}[\mathrm{GA}]_i\Big), \tag{3.8}$$

where α denotes the ratio between the GA synthesis rates in the elongation zone to meristem and we have simplified the last term using equation (1.1). Here, the first term on the right-hand-side represents synthesis and degradation, whereas the second term represents dilution due to expansion of the vacuole.

Equations (3.6) and (3.8) are coupled to (1.1) and are solved using matlabs ode solver *ode45* in the time intervals between the division events.

3.3 Specifying the spatially varying synthesis rate

To investigate our hypothesis that the higher GA levels exhibited in the elongation zone reflects an increase in GA synthesis rate, we specify a synthesis rate that increases with distance from the QC, x, of the form

$$\sigma(x) = \sigma_{QC} + \frac{\alpha x^n}{\xi + x^n}, \tag{3.9}$$

where constant σ_{QC} represents the synthesis rate at the QC, α and ξ are constants, and n is a natural number. With this Hill function form, the synthesis rate varies smoothly between a low value and a high value, with a more step-like form for higher values of n, occurring at ξ microns from the QC.

4 Predicting the nlsGPS1 distribution

Once the model has predicted the GA distribution, we use the following relationship suggested in Rizza et al, 2017 to calculate the corresponding distribution of nlsGPS1

$$nlsGPS1 = a + \frac{B[\mathrm{GA}]^h}{K_d^h + [\mathrm{GA}]^h}; \tag{4.1}$$

parameter values are given in Table S1.

5 Simulating the exogeneous GA experiments

To simulate the exogeneous GA experiments, we extended our governing equations to include an extra term representing the rate at which the exogeneous GA enters the cells. We suppose that the rate at which GA enters the root is proportional to the external GA concentration $[\mathrm{GA}]_{dose}$, with the constant of proportionality referred to as the permeability of the root to GA, P_{root}. Thus, the governing equations (3.6) and (3.8) become

$$\frac{d[\mathrm{GA}]_i}{dt} = [\mathrm{GA}]_{dose} P_{root} + \gamma(\sigma - \beta[\mathrm{GA}]_i) - \mathrm{RER}_m[\mathrm{GA}]_i \quad \text{for meristem cells,} \tag{5.1}$$

$$\frac{d[\mathrm{GA}]_i}{dt} = [\mathrm{GA}]_{dose} P_{root} + \frac{1}{L_{mi} + \kappa(l - L_{mi})}\left(\gamma L_{mi}(\sigma - \beta[\mathrm{GA}]_i) - \kappa l_i \mathrm{RER}_{EZ}[\mathrm{GA}]_i\right), \quad \text{for elongation zone cells.} \tag{5.2}$$

We considered several cases for the form of the permeability, P_{root}. Firstly we considered a constant permeability, see Fig 3D and Fig S8A.

Secondly, we considered the permeability to depend on the distance from the QC, x; considering the form

$$P_{root}(x) = P_{QC} + \frac{\zeta x^2}{\lambda^2 + x^2}, \tag{5.3}$$

where P_{QC}, ζ and λ are constants (see Fig 3E,F, Fig S8B). This form represents a permeability that varies smoothly between a low value close to the QC and a higher value for cells far from the QC.

6 Parameter considerations

6.1 Specifying the synthesis and degradation parameters via parameter surveys and comparison with sensor data

The model parameters were specified from our own data or estimates in the literature where possible (as summarised in Table S1). Parameters related to the growth dynamics were estimated from our own growth data (Figure S2, Table S1), parameters in the relationship between the GA concentration and sensor data were characterised in Rizza et al 2017, and parameters related to the pH and dissociation constant were available in the literature (Kramer, 2004; Kramer 2006; Table S1). However, the appropriate spatial distribution of the GA synthesis rate and the value of the GA degradation rate are unknown as these will depend on the in vivo activity levels of the metabolism enzymes. We investigated parameter space by performing a simple parameter survey, considering uniform distributions of the parameter values that characterise the GA synthesis rate distribution:

$$\alpha \in [0.0002, 0.0004, \cdots, 0.002], \xi \in [25, 50, \cdots, 250]\ \sigma_{QC} = [0.00001, 0.00002, \cdots 0.0001], \tag{6.1}$$

and calculating the difference between the predicted and observed sensor data via

$$f = \sum_{i=1}^{N} (\text{Model}(x_i) - \text{Data}(x_i))^2 \tag{6.2}$$

where $\text{Data}(x_i)$ denotes the sensor data which comprises N measurements at distance x_i from the QC (for $i = 1, 2, N$). The predicted sensor data at x_i is denoted $\text{Model}(x_i)$ and is calculated from the model prediction (using linear interpolation over space). The parameter survey was performed for two values of the Hill function co-efficient: $n = 2$ which enables us to consider a smoothly increasing synthesis rate and $n = 10$ which provides an example of a step-like increase in synthesis rate. Furthermore, we consider two values of the GA degradation rate (since the *ga2oxq* sensor data motivates the choice of the small degradation rate, as discussed in the main text). In each case, we selected the parameter set $[\alpha, \xi, \sigma_{QC}]$ which minimises f (6.2). In the case of $n = 2$, $\beta = 0.05$, the σ_{QC} parameter obtained was on the lower boundary of the investigated parameter space, therefore the parameter survey was rerun to investigate a space with smaller $\sigma_{QC} \in [0.000002, 0.000004, \cdots, 0.00002]$. The resulting parameter sets are give in Table S2.

6.2 Specifying the permeability parameters via parameter surveys and comparison with sensor data

In the simulations of the exogeneous GA, similar parameter surveys were performed. For the case of constant permeability, we estimated the single parameter, $P_{root} = P_{QC}$ (setting $\lambda = \zeta = 0$), whereas for the spatially varying permeability, we estimated the values of the three parameters in the permeability distribution (5.3), $[\zeta, \lambda, P_{QC}]$. In each case, we considered the following uniform distributions for these parameters:

$$\zeta \in [1, 2, \cdots, 10], \quad \lambda \in [50, 100, \cdots, 500], \quad P_{QC} = [0.05, 0.1, \cdots 0.5]. \tag{6.3}$$

We selected the parameter set (given in Table S1) which minimises the difference between the model predictions and data 20 minutes after the application of exogeneous GA (by calculating f as specified in (6.2)). We also investigated a spatially varying permeability with a higher Hill co-efficient equal to 10 to represent a more step-like increase in permeability; however, estimating the parameters $[\zeta, \lambda, P_{QC}]$ and calculating the minimum f, (6.2), in each case, we concluded that there is better agreement between model predictions and data with the lower Hill co-efficient of 2 (as stated in (5.3)).

6.3 Assessing the influence of stochasticity on the model predictions

The model is stochastic, due to stochasticity in cell division times. The synthesis rate of a cell is specified based on the distribution given in using the position of the cell centre. Hence if this cell divides, the daughter cells will have different synthesis rates, based on the positions of their respective cell centres. Thus, the stochasticity in division events affects GA synthesis and hence the predicted GA distribution. This process only has a minor effect, however, and we find very little difference in the predicted GA distribution for different simulation replicates (see Figure S5E). The results presented throughout are an average from 20 replicates (where the predicted GA and nlsGPS1 distributions are interpolated to a uniform grid prior to averaging).

6.4 Assessing the influence of the growth parameters

Although the growth parameters were estimated from our own measurements or the literature, we investigate here how the choice of these growth parameters affect the model predictions.

Increasing the RER specified for the meristem or elongation zone increases the rate of GA dilution in the respective zone, as well as increasing the rate at which cells traverse the developmental zones. As one would expect, with a larger meristematic RER, we predict lower GA concentrations throughout the growth zone (Figure S5A), whereas a larger elongation-zone RER results in lower predicted GA concentrations in the elongation zone (Figure S5B).

The proportion of meristematic cell volume that is cytoplasm, γ, has been estimated in dividing cells in Arabidopsis as approximately 0.7 (Willis et al, 2016) (although we note that this value was measured in

the shoot apical meristem, and as far as we are aware this has not been measured in the Arabidopsis root meristem). Since key GA metabolism enzymes reside in the cytoplasm (Olszewski et al, 2002), our equations show that γ influences the effective magnitude of synthesis and degradation. The model predicts that a lower γ would result in a small decrease in GA concentration throughout the elongation zone (Figure S5C).

Another key parameter is the ratio between the GA concentration in the vacuole to the cytoplasm, κ. Here, we follow Band et al, 2012 in assuming that only protonated GA can cross the tonoplast into the vacuole, resulting in a vacuolar GA concentration that is approximately 1/15 of the cytoplasmic concentration. However, it is possible that GA transporters may be present and enable anionic GA to enter the vacuole, a possibility that is suggested by the recent observation of substantial fluorescence-tagged GA in the vacuole (Shani et al, 2013), suggesting larger values of κ may be possible. Increasing κ would increase the rate of dilution in the elongation zone where cell elongation is thought to be predominantly due to vacuolar expansion. Thus, as expected, with larger values of κ the model predicts lower GA concentrations in the elongation zone (Figure S5D). We see that with κ greater than around 0.2, the model predicts a peak in the GA concentration in the centre of the elongation zone with the GA concentration then reducing as cells move towards the mature zone. We note that this peak is not observed in the nlsGPS1 data suggesting that should higher values of κ be shown to be appropriate in the future even higher elongation-zone synthesis would be required to counteract the effect of dilution.

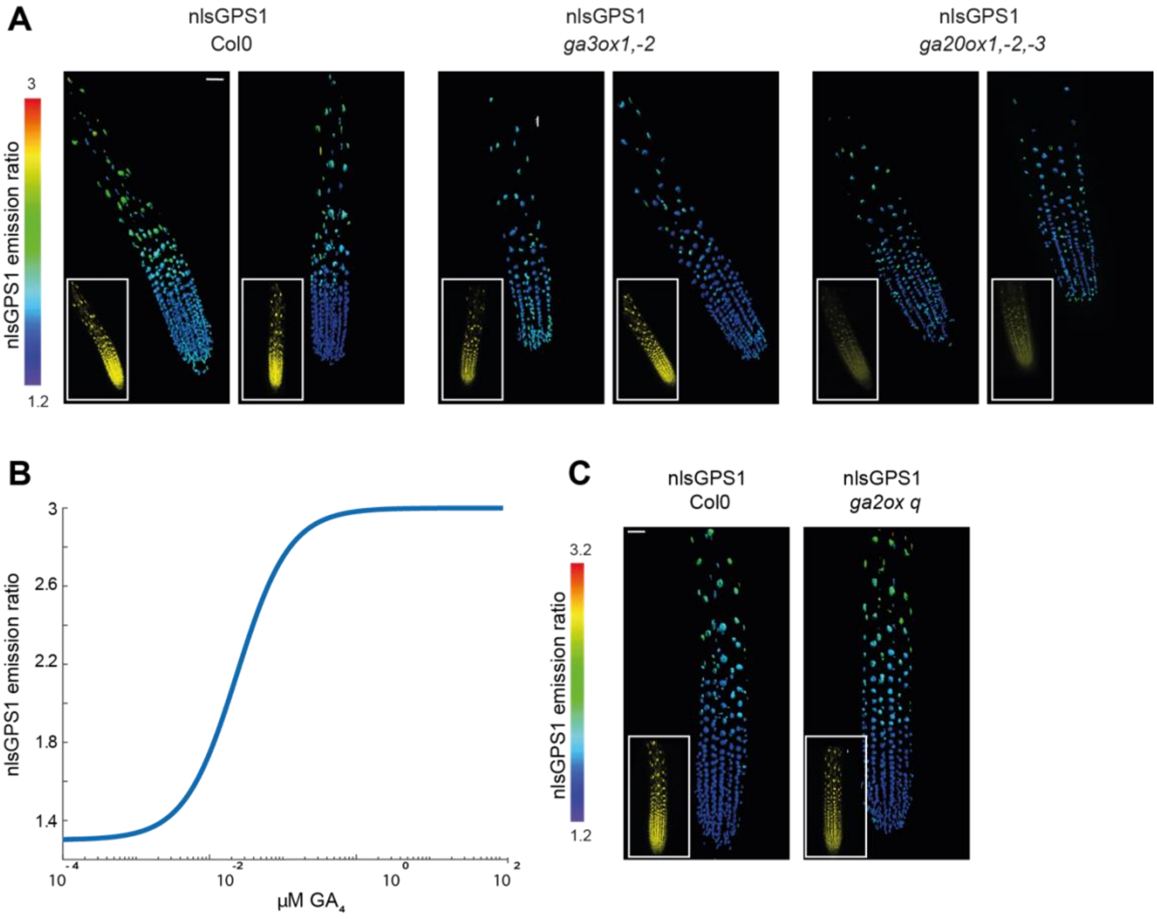

Fig. S1. nlsGPS1 in mutant backgrounds. (A) 3D images of nlsGPS1 emission ratios and YFP fluorescence of roots four days post sowing in wild-type Col0 or GA biosynthetic mutant *ga3ox1, ga3ox2* and *ga20ox1, ga20ox2, ga20ox3* backgrounds. (B) Relationship between GA concentration and nlsGPS1 emission ratio based on the nlsGPS1 GA_4 titration data in (6). (C) 3D images of nlsGPS1 emission ratios and YFP fluorescence of roots four days post sowing in wild-type Col0 or GA catabolic quintuple mutant *ga2ox1, ga2ox2, ga2ox3, ga2ox4, ga2ox6* (*ga2ox q*). Scale bar: 30 µm.

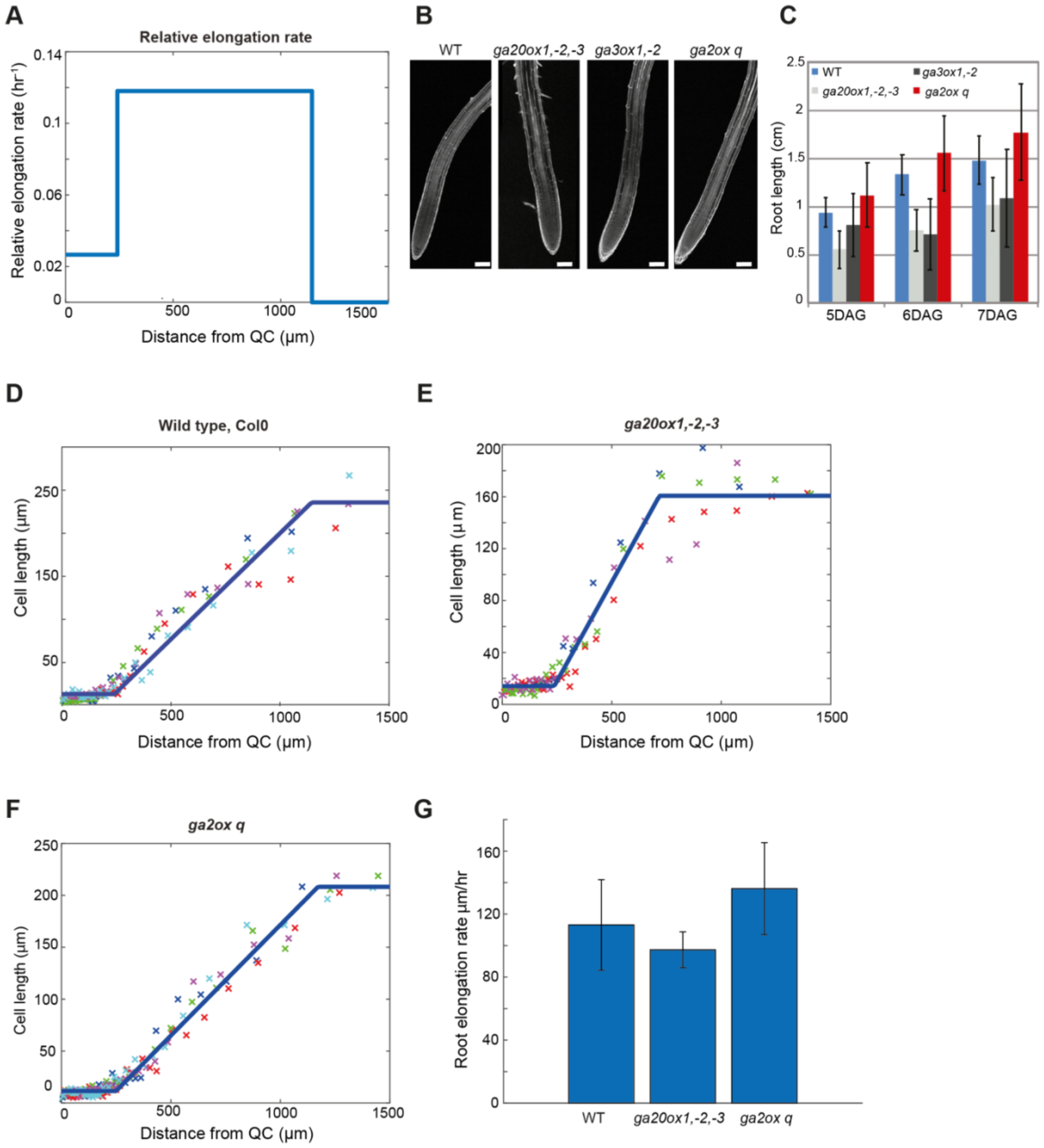

Fig. S2. Model growth parameters. (A) Cellular relative elongation rates for Col0 prescribed in the mathematical model; this form was calculated from the data shown in panels C, D, and G as described in the Supplementary Information Text. (B) Representative images of PI-stained roots of WT and mutant background. Scale bar: 30μm (C) Root length of Wild-type Col0 and mutant backgrounds after 5, 6, and 7 DAG. (D - F) Data and fitted distributions for the spatial profile of the cell lengths in the growth zones of wild-type (D), *ga20ox1, ga20ox2, ga20ox3* triple mutant background (E), and *ga2ox* quintuple mutant background (F) (see Supplementary Information Text and Table S1 for further details). (G) Root elongation rate for wild-type and GA metabolic mutant backgrounds calculated from the root length data shown in panel C.

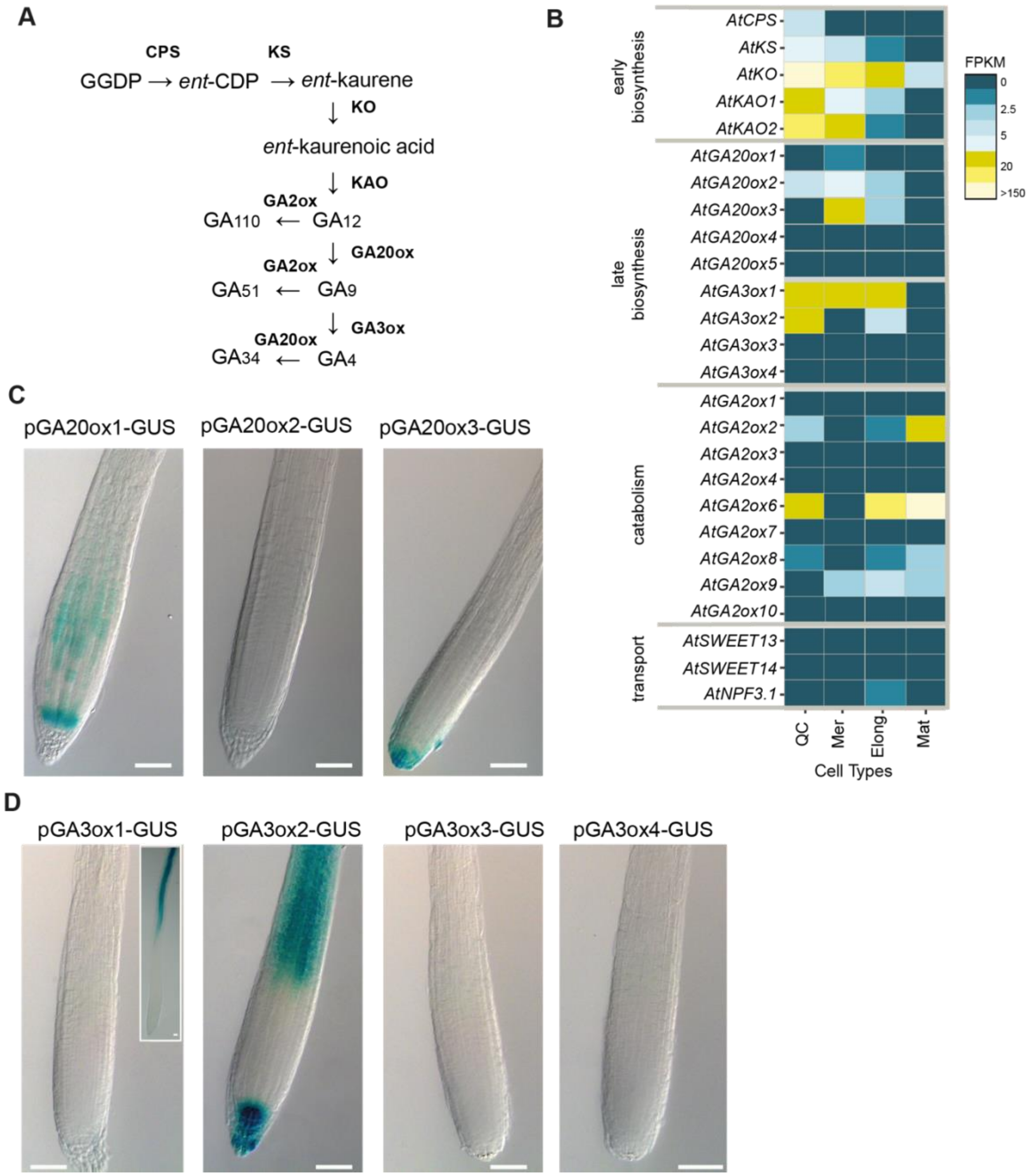

Fig. S3. Expression pattern of GA metabolic enzymes and transporters. (A) Schematic representation of the GA metabolic pathway. (B) Heat map of the expression level GA metabolic enzymes (biosynthesis and catabolism) and GA transporters in different regions of roots (QC - quiescent center, Mer - root meristem, Elong - elongation zone, Mat - maturation zone). Data is from Li, et al. (9). (C) Five-day-old post sowing roots expressing *AtGA20ox1*, *AtGA20ox2*, *and AtGA20ox3* gene promoter in fusion with the GUS reporter. Lines are from Plackett et al. (10) (D) Five-day-old post sowing roots expressing *AtGA3ox1, AtGA3ox2, AtGA3ox3 and AtGA3ox4* gene promoter in fusion with the GUS reporter. Small panel of pGA3ox1-GUS root shows the expression of *AtGA3ox1* in maturation zone. Lines are from Mitchum et al. (11). All the lines are transcriptional fusion (TC) of GA biosynthesis promoter to GUS, except for pGA20ox1-GUS and pGA3ox4-GUS where translational fusion (TL) lines were used. Scale bar = 50μm.

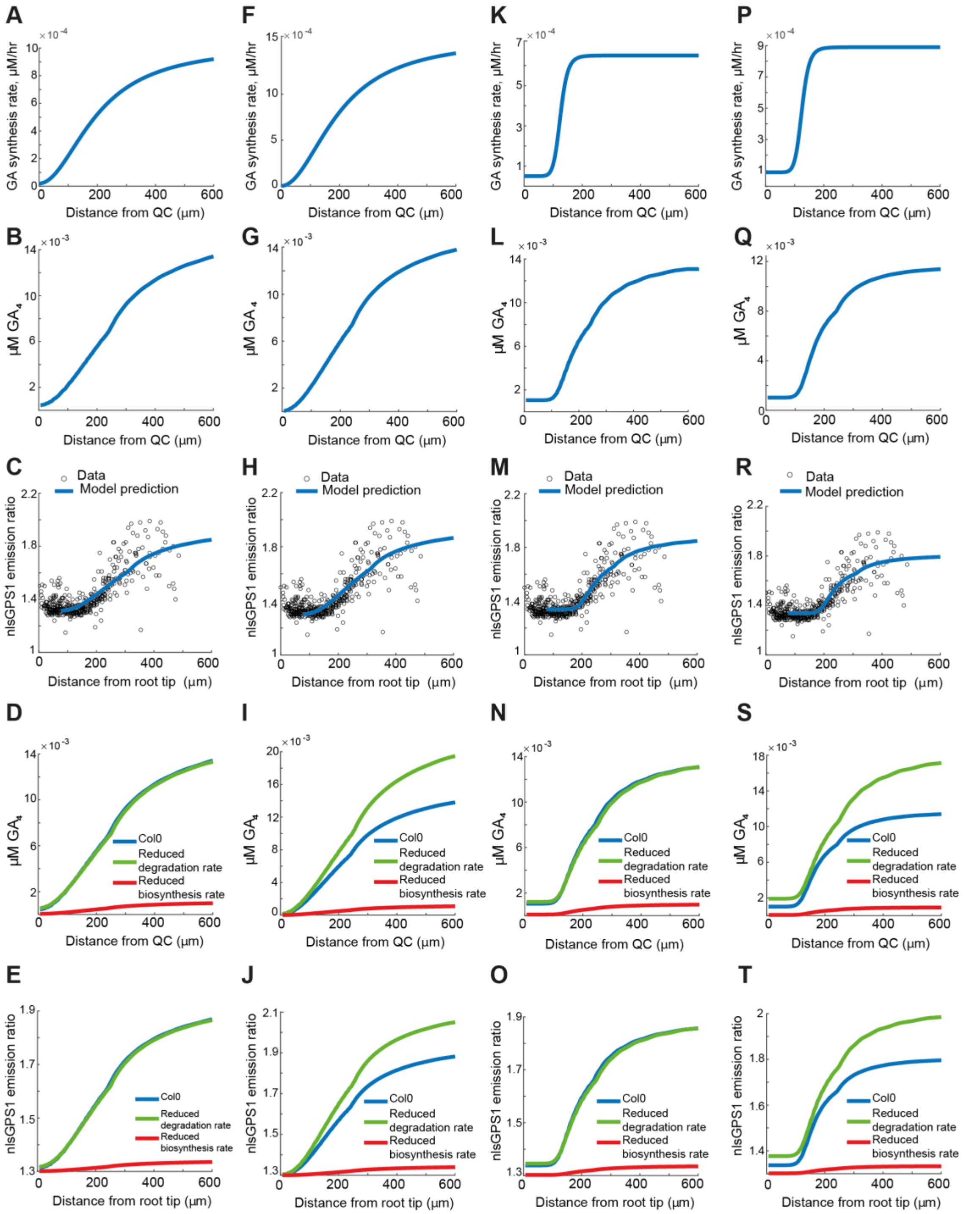

Fig. S4. Model predictions with 'best-fit' parameter sets obtained from the parameter survey. (A-E) Case n=2, β=0.01. (F-J) Case n=2, β=0.05. (K-O) Case n=10, β=0.01. (P-T) Case n=10, β=0.05. The remaining parameter values are given in Tables S1 and S2. (A,F,K,P) Distribution of GA synthesis rates obtained from parameter survey. (B, G, L, Q) Wild-type predictions of GA distribution. (C, H, M, R) Wild-type predictions of nlsGPS1 distribution with nlsGPS1 data. (D, E, I, J, N, O, S, T) Predictions of GA and nlsGPS1 for wild-type, *ga2oxq* (reduced degradation rate, assumed to be 10% of the wild-type value) and *ga20ox1, ga20ox2, ga20ox3* (reduced synthesis rate, assumed to be 10% of the wild-type value).

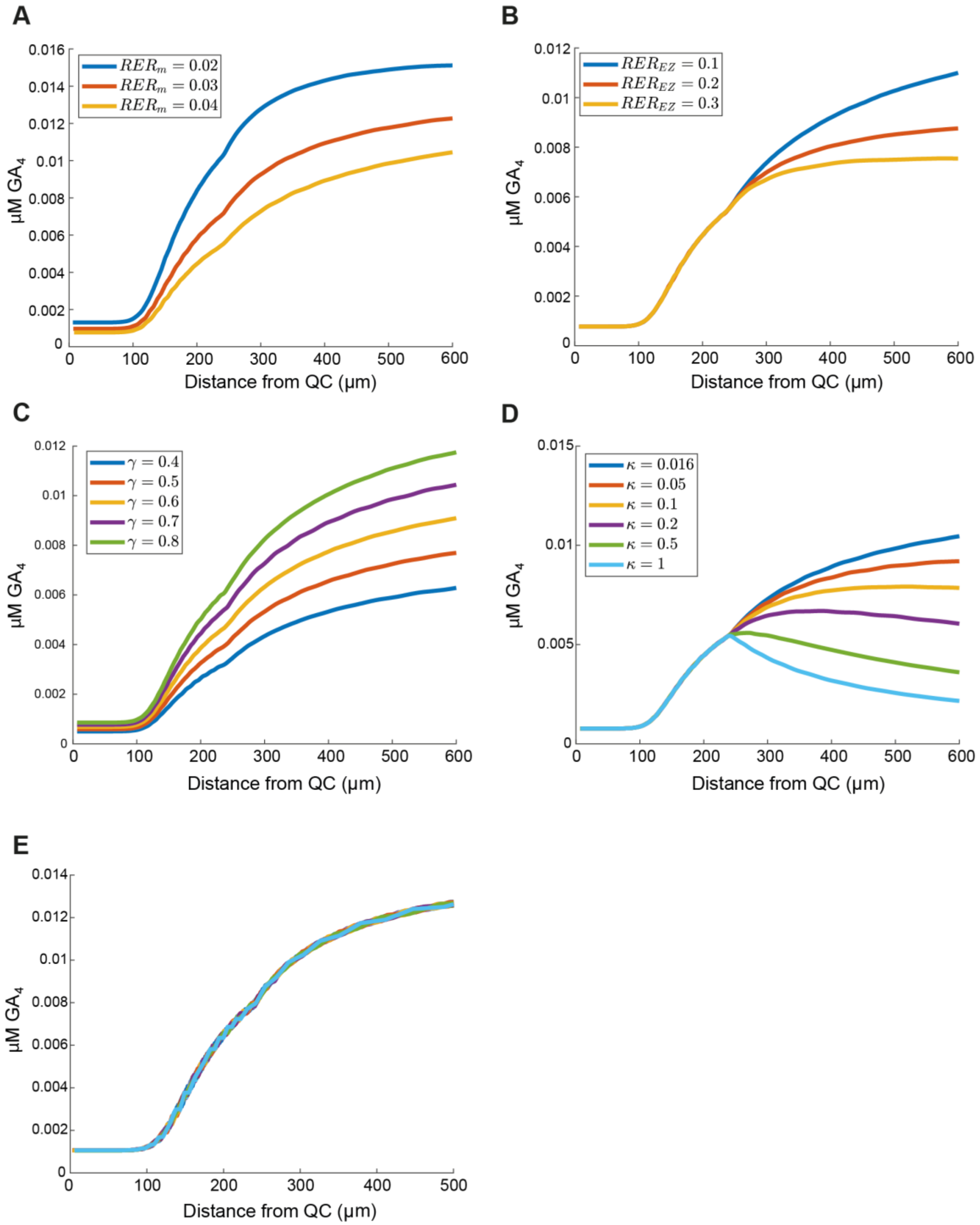

Fig. S5. Model parameter testing. (A-D) Effect of model parameter values on the predicted GA distribution. (A) Relative elongation rate in the meristem (RER_m), (B) Relative elongation rate in the elongation zone (RER_{EZ}), (C) Proportion of meristematic cell volume that is cytoplasm (γ), (D) Ratio between the GA concentration in the vacuole to the cytoplasm (κ). (E) Model predictions for GA distribution for 20 different replicates of the simulations show that the stochasticity has a small effect on the model predictions.

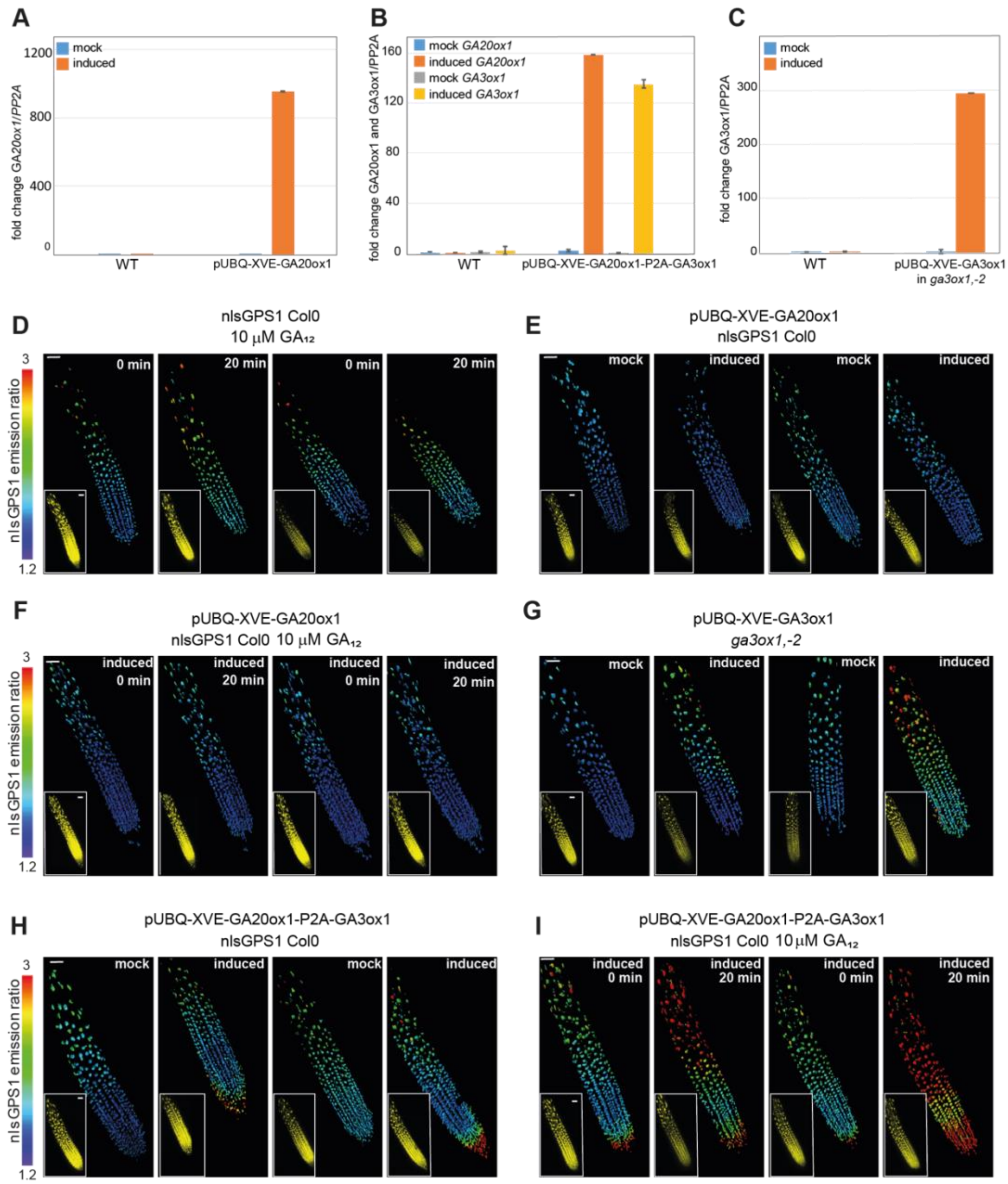

Fig. S6. GA enzyme induction and precursor treatment. (A-C) qPCR analysis of GA enzyme expression in WT and inducible GA enzyme transgenic lines. Induction was 5 µM 17-β-estradiol for 24 hrs while mock induction was 0.2% DMSO. Reference gene: PP2AA3 (At1g13320). (A) *AtGA20ox1* expression in pUBQ-XVE-AtGA20ox1 transgenic line. (B) *AtGA20ox1* and *AtGA3ox1* expression in pUBQ-XVE-AtGA20ox1-P2A-AtGA3ox1 transgenic line. (C) *AtGA3ox1* expression in pUBQ-XVE-AtGA3ox1 transgenic line in *ga3ox1, ga3ox2* mutant background. (D-I) Other representative 3D images of nlsGPS1 emission ratios and YFP fluorescence of roots five days post sowing. (D, F, I) Before (0 min) and 20 minutes after treatment with 10 µM GA_{12}. (E-I) 24 hrs after 5 µM 17-β-estradiol (induced) or 0.2% DMSO (mock) as indicated. (D) Wild-type Col0. (E-F) pUBQ-XVE-AtGA20ox1 line. (G) pUBQ-XVE-AtGA3ox1 line in *ga3ox1, ga3ox2* mutant background. (H) pUBQ-XVE-AtGA20ox1-P2A-AtGA3ox1 line. Scale bar: 30 µm.

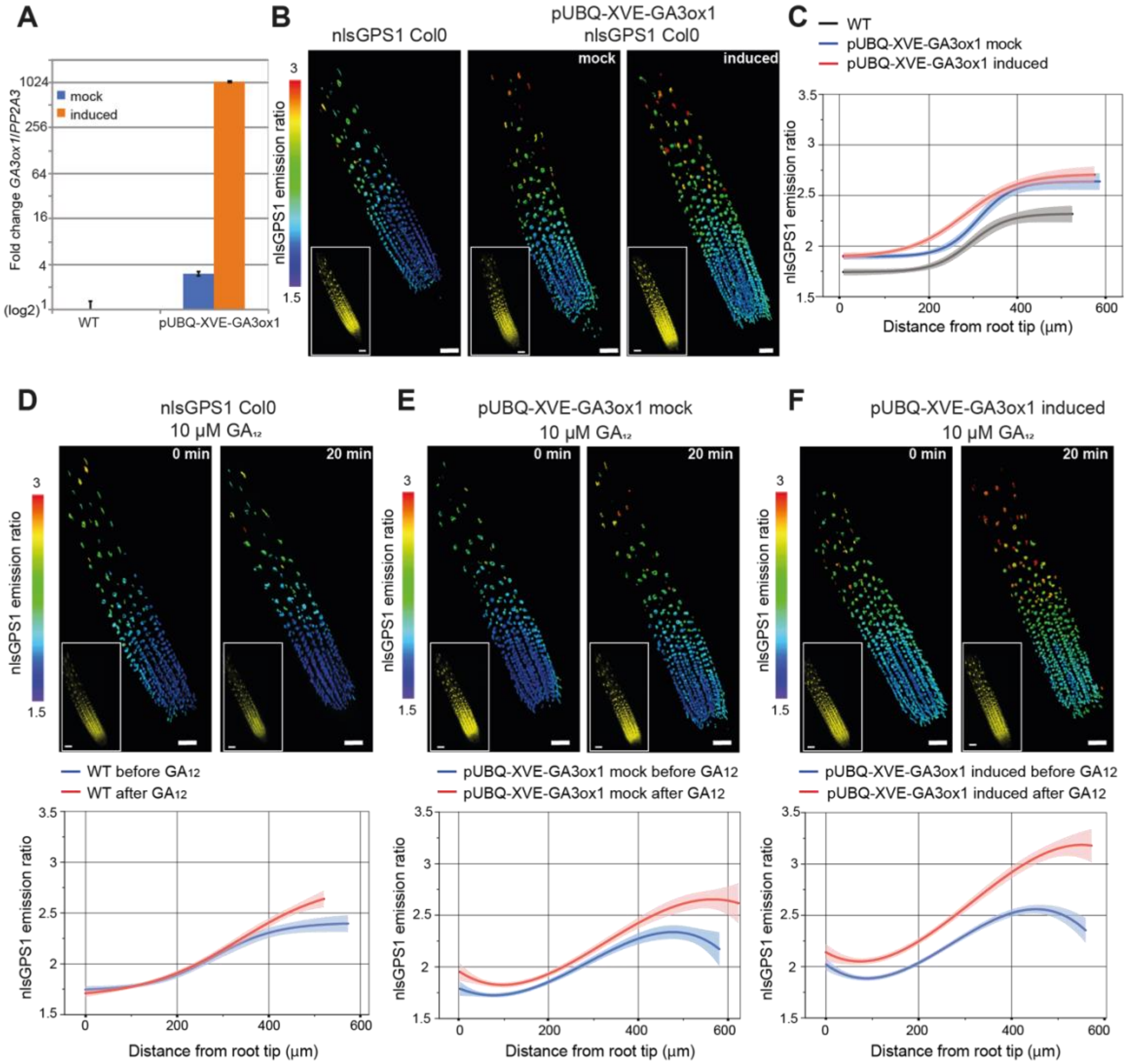

Fig. S7. GA3ox1 enzyme induction and precursor treatment. (A) qPCR analysis of *AtGA3ox1* expression in WT and pUBQ-XVE-AtGA3ox transgenic lines. Induction was 5 μM 17-β-estradiol for 24 hrs while mock induction was 0.2% DMSO. Reference gene: PP2AA3 (At1g13320). (B) Representative 3D images of nlsGPS1 emission ratios and YFP fluorescence of WT and pUBQ-XVE-AtGA3ox1 roots five days post sowing 24 hrs after 5 μM 17-β-estradiol (induced) or 0.2% DMSO (mock). (C) Curves of best fit and 95% confidence intervals are computed in R using local polynomial regression (Loess) via ggplot, with smoothing parameter span=0.75. (D, E, F) Before (0 min) and 20 minutes after treatment with 10 μM GA_{12}. Representative 3D images (upper) and curves of best fit with 95% confidence intervals (lower). (D) Wild-type Col0. (E) pUBQ-XVE-AtGA3ox1 line mock. (F) pUBQ-XVE-AtGA3ox1 line induced. Scale bar: 30 μm.

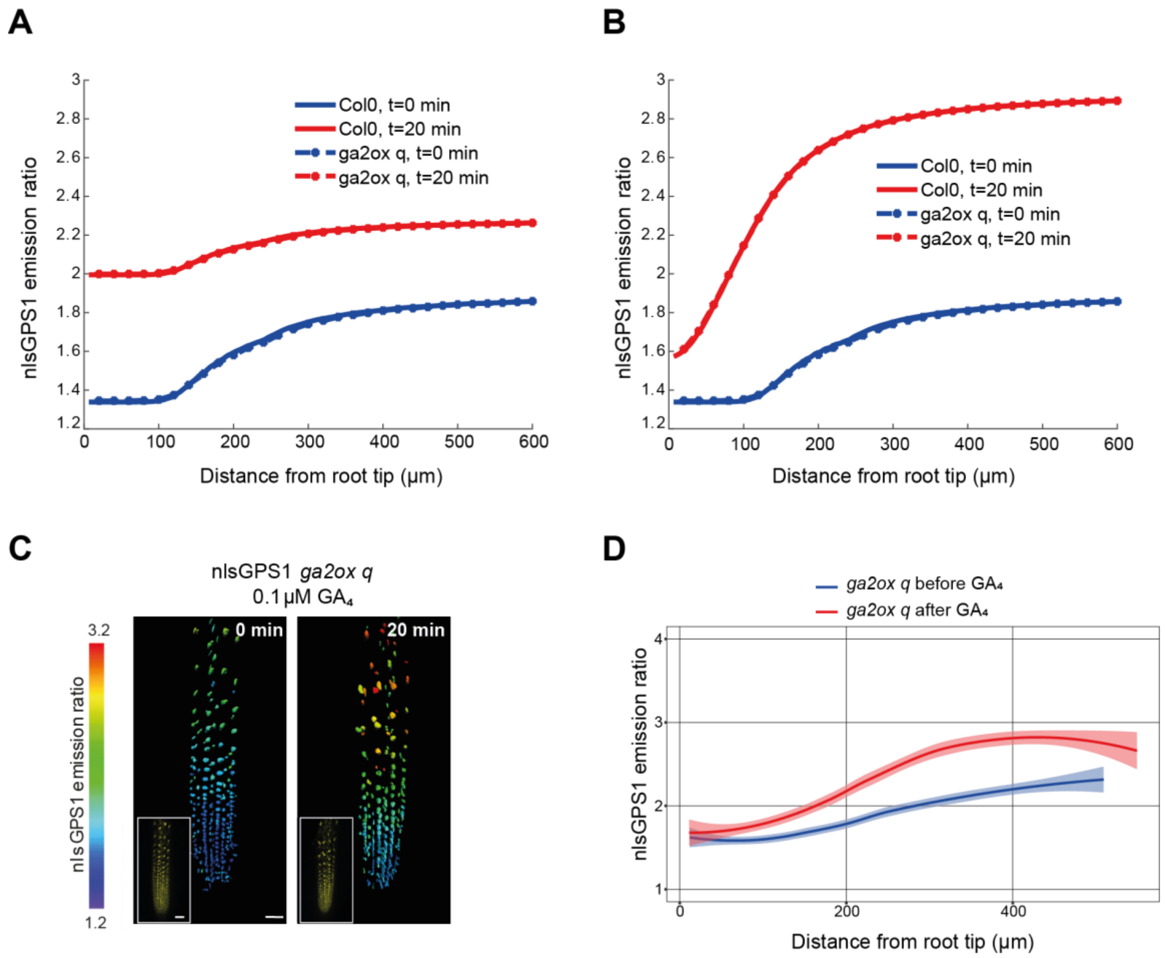

Fig. S8 Catabolism and permeability model predictions and testing. (A) Predictions of nlsGPS1 emission ratios for wild-type versus *ga2oxq* (reduced degradation rate, assumed to be 10% of the wild-type value) with constant permeability. (B) Predictions of nlsGPS1 emission ratios for wild-type versus *ga2ox q* (reduced degradation rate, assumed to be 10% of the wild-type value) with gradually increasing permeability as shown in Fig 3E. (C) 3D images of nlsGPS1 emission ratios and YFP fluorescence of *ga2ox q* roots before and 20 minutes after 0.1 µM GA_4. (D) nlsGPS1 emission ratios for individual nuclei of *ga2ox q* mutant in relation to distance in µm from the root tip, before and after treatment with GA_4. Curves of best fit and 95% confidence intervals are computed in R using local polynomial regression (Loess) via ggplot, with smoothing parameter span=0.75. Scale bar: 30 µm.

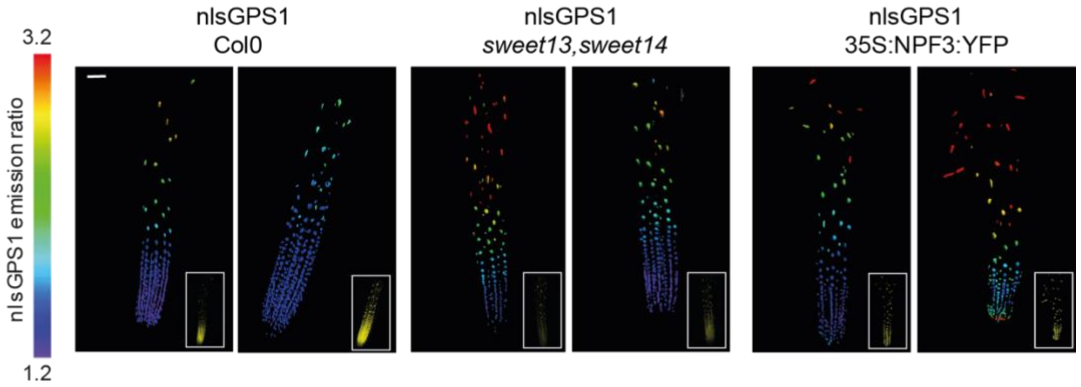

Fig. S9. nlsGPS1 in transporter mutants. Other representative 3D images of nlsGPS1 emission ratios and YFP fluorescence of roots five days post sowing in wild-type, *sweet13, sweet14* double mutant or 35S:NPF3:YFP transgenic line backgrounds. Scale bar: 30 µm.

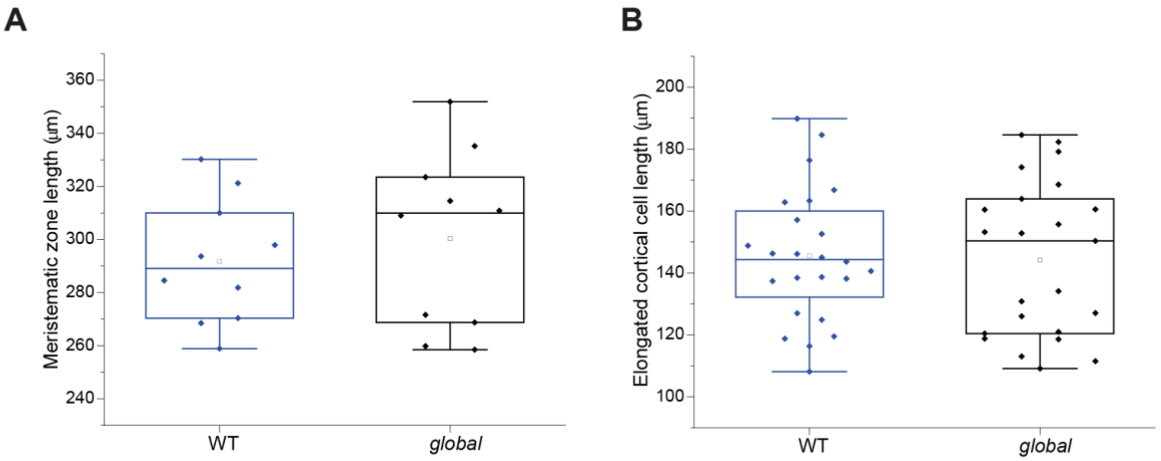

Fig. S10. Root growth in *global* mutant vs wild-type Ler. (A) Beeswarm and box plot of meristematic zone length of five-day-old roots of nlsGPS1 in Ler vs *global* mutant. (B) Beeswarm and box plot of cortical cell (950 µm from QC) length of five-day-old roots of nlsGPS1 in Ler vs *global* mutant.

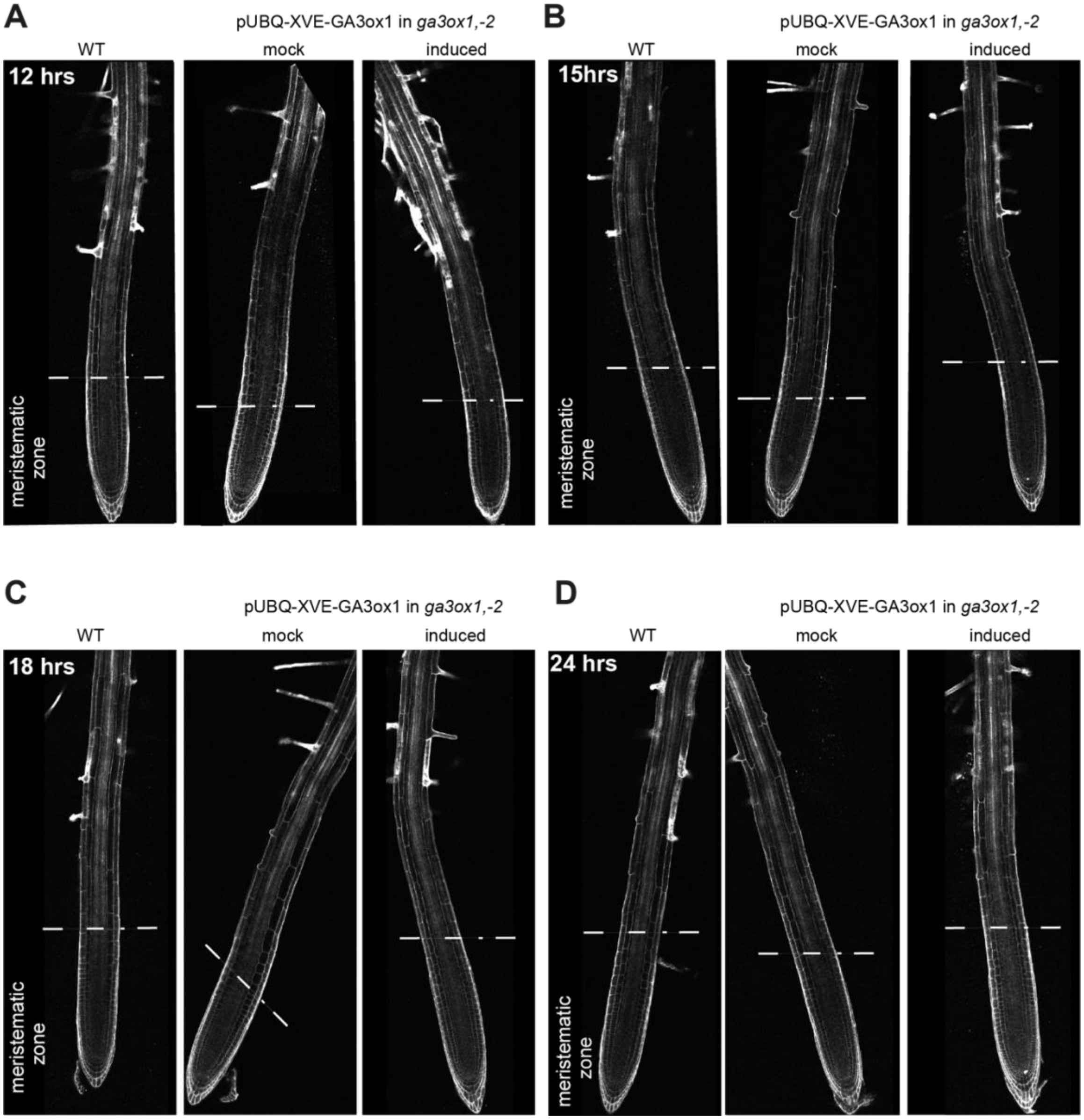

Fig. S11. Root growth complementation time course. Representative images of propidium iodide (PI) staining nlsGPS1 roots shown in Figure 6 in wild-type or pUBQ-XVE-AtGA3ox1 line in *ga3ox1, ga3ox2* mutant background, after twelve, fifteen, eighteen and twenty-four hours induction with of 5 μM 17-β-estradiol or mock induction (0.2% DMSO).

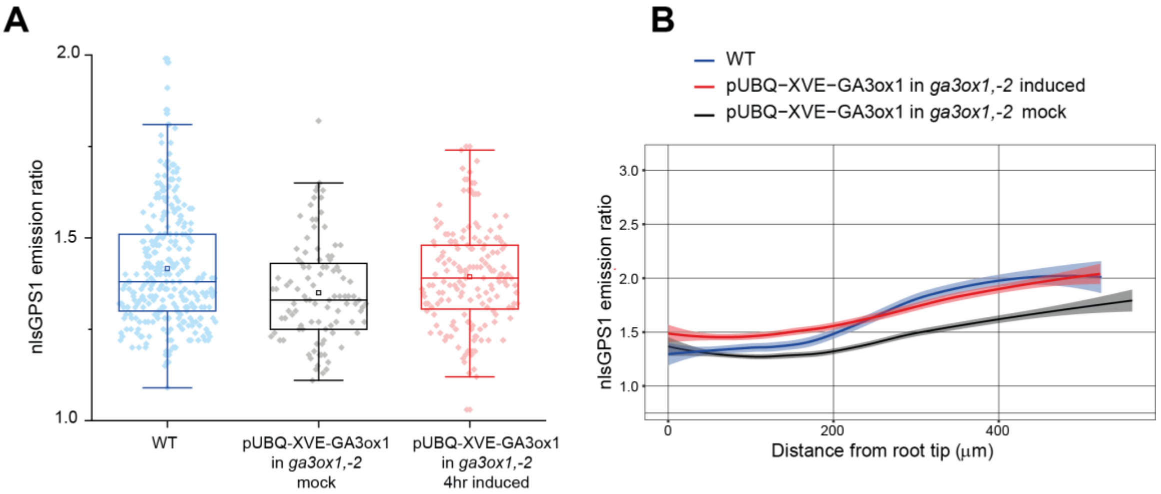

Fig. S12. Complementation of GA levels in *ga3ox1, ga3ox2* mutant. (A) Beeswarm and box plots of nlsGPS1 emission ratios for individual nuclei of elongated cells (350µm from root tip) of four-day-old roots (*n* = 5). Wild-type or pUBQ-XVE-AtGA3ox1 inducible line in *ga3ox1, ga3ox2* mutant background are shown after four hours induction with of 5 µM 17-β-estradiol or mock induction (0.2%DMSO) (B) nlsGPS1 emission ratios in relation to distance in µm from the root tip for representative roots of wild-type or pUBQ-XVE-AtGA3ox1 inducible line in *ga3ox1, ga3ox2* mutant backgrounds after twelve hours induction with of 5µM 17-β-estradiol or mock induction (0.2% DMSO) shown. Curves of best fit and 95% confidence intervals are computed in R using local polynomial regression (Loess) via ggplot, with smoothing parameter span=0.75.

Table S1. Table of final model parameters.

Parameter	Description	Value	Reference
pH_{vac}	pH in the vacuole	5.8	Kramer 2004
pH_{cyt}	pH in the cytoplasm	7.6	Kramer 2004
pK	Dissociation constant for GA_4	4.2	Kramer 2006
κ	Ratio between vacuole and cytoplasm concentrations	0.0162	Calculated from pH_{vac}, pH_{cyt} and pK
σ_{QC}	Synthesis rate at the QC	0.00005 μM /hr	Chosen via parameter survey to obtain agreement between model predictions and data
α	Parameter in the GA synthesis rate formula	0.0006 μM /hr	Chosen via parameter survey to obtain agreement between model predictions and data
ξ	Parameter in the GA synthesis rate formula	125 μm	Chosen via parameter survey to obtain agreement between model predictions and data
n	Parameter in the GA synthesis rate formula	10	Chosen via parameter survey to obtain agreement between model predictions and data
β	Degradation rate	0.01 /hr	Chosen to obtain agreement between model predictions and data for *ga2ox*
γ	Proportion of the cell volume that is cytoplasm in the meristem	0.7	Willis et al. 2016
a	Parameter in GA to nlsGPS1 relationship	1.3	Rizza et al. 2017
Bmax	Parameter in GA to nlsGPS1 relationship	1.7	Rizza et al. 2017
h	Parameter in GA to nlsGPS1 relationship	1.21	Rizza et al. 2017
Kd	Parameter in GA to nlsGPS1 relationship	0.0237 μM	Rizza et al. 2017
Tcse	Standard error of the time interval between successive cell division	0.7 hr	Beemster and Baskin, 1998

Model parameters obtain from growth phenotyping (see data in Fig S3):

Line	Parameter	Description	Value
Wild type	x_m	Length of meristem	241 μm
	x_{EZ}	Length of elongation zone	904 μm
	RER_m	Relative elongation rate in the meristem	0.027 /hr

	RER_{EZ}	Relative elongation rate in the elongation zone	0.12 /hr
ga20ox	x_m	Length of meristem	237 μm
	x_{EZ}	Length of elongation zone	481 μm
	RER_m	Relative elongation rate in the meristem	0.036 /hr
	RER_{EZ}	Relative elongation rate in the elongation zone	0.18 /hr
ga2ox	x_m	Length of meristem	249 μm
	x_{EZ}	Length of elongation zone	923 μm
	RER_m	Relative elongation rate in the meristem	0.030 /hr
	RER_{EZ}	Relative elongation rate in the elongation zone	0.14 /hr

Model parameters related to the simulations of exogeneous GA

Parameter	Description	Value
GA_{dose}	Exogeneous GA concentration	0.1 μM
P_{root}	Constant permeability used in Fig 3D	0.5 /hr
P_{QC}	Permeability at QC used in Fig 3F	0.15 /hr
ζ	Parameter in permeability function in Fig 3F, see Supplementary text.	9 /hr
λ	Parameter in permeability function in Fig 3F, see Supplementary text.	400 μm

Table S2: Table of parameter values obtained from parameter survey (see Supplementary Information Text for details).

Case		Parameters obtained from parameter survey			Difference between predictions and data
n	β	σ_{QC}	α	ξ	f
2	0.01	0.00002	0.001	200	3.31
2	0.05	0.000004	0.0016	200	3.28
10	0.01	0.00005	0.0006	125	3.04
10	0.05	0.00009	0.0008	125	2.95

Table S3. Primer list

Cloning primer list

AtGA20ox1	FW: GGGGACAAGTTTGTACAAAAAAGCAGGCTTCATGGCCGTAAGTTTCGTAACA
	RV: GGGGACCACTTTGTACAAGAAAGCTGGGTGTTAGATGGGTTTGGTGAGCC
AtGA3ox1	FW: GGGGACAAGTTTGTACAAAAAAGCAGGCTTCATGCCTGCTATGTTAACAGATGT
	RV: GGGGACCACTTTGTACAAGAAAGCTGGGTGTCATTCTTCTCTGTGATTTCTAATC
AtGA20ox1-P2A	FW: GGGGACAAGTTTGTACAAAAAAGCAGGCTTCATGGCCGTAAGTTTCGTAACA
	RV + P2A: CCTCCACGTCTCCAGCCTGCTTCAGCAGGCTGAAGTTAGTAGCTCCGCTTCCGATGGGTTTGGTGAGCCA
P2A AtGA3ox1	FW: GCCTGCTGAAGCAGGCTGGAGACGTGGAGGAGAACCCTGGACCTATGCCTGCTATGTTAACAGATGT
	RV: GGGGACCACTTTGTACAAGAAAGCTGGGTGTCATTCTTCTCTGTGATTTCTAATC

Quantitative Real-Time PCR (qPCR) primer list

PP2AA3	AGACAAGGTTCACTCAATCCGTG
	CATTCAGGACCAAACTCTTCAGC
AtGA20ox1	GATCCATCCTCCACTTTAGA
	GTGTATTCATGAGCGTCTGA
AtGA3ox1	CCATTCACCTCCCACACTCT
	GCCAGTGATGGTGAAACCTT

Movie S1 (separate file). Time-course of GA treatments at standard then low pH of nlsGPS1 roots growing in Rootchip18S. Imaging was acquired every 5 minutes for 2 hours and 30 minutes with the following intervals: 20 minutes of mock solution (pH 5.7), 20 minutes of 50 nM GA_4 at pH 5.7, 30 minutes of mock solution, 20 minutes of 50 nM GA_4 at pH 4.5, and 60 minutes of mock solution.

Movie S2 (separate file). Time-course of GA treatments at low then standard pH of nlsGPS1 growing in Rootchip18S. Imaging was acquired every 5 minutes for 2 hours and 15 minutes with the following intervals: 20 minutes of mock solution (pH 5.7), 20 minutes of 50 nM GA_4 at pH 4.5, 30 minutes of mock solution, 20 minutes of 50 nM GA_4 at pH 5.7, and 45 minutes of mock solution.

Movie S3 (separate file). Time-course of mock treatments at low pH of nlsGPS1 growing in Rootchip18S. Imaging was acquired every 5 minutes for 1 hour and 40 minutes with the following intervals: 20 minutes of mock solution (pH 5.7), 60 minutes of mock solution at pH 4.5, and 20 minutes of mock solution.

SI References

1 L. R. Band, *et al.*, Growth-induced hormone dilution can explain the dynamics of plant root cell elongation. *Proc. Natl. Acad. Sci.* 109, 7577–7582 (2012).

2 G.T.S. Beemster and T. I. Baskin. Analysis of cell division and elongation underlying the developmental acceleration of root growth in Arabidopsis thaliana. *Plant Physiol.* 116: 1515–1526 (1998).

3 E. M. Kramer. PIN and AUX/LAX proteins: their role in auxin accumulation. *Trends Plant Sci.*, 9:578–582 (2004).

4 E. M. Kramer. How Far Can a Molecule of Weak Acid Travel in the Apoplast or Xylem? *Plant Physiology*, 141:1233–1236 (2006).

5 N. Olszewski, T. P. Sun, F. Gubler, Gibberellin signaling: Biosynthesis, catabolism, and response pathways. *Plant Cell* (2002)

6 Rizza, A. Walia, V. Lanquar, W. B. Frommer, and A. M. Jones. In vivo gibberellin gradients visualized in rapidly elongating tissues. *Nature Plants*, 3: 803-813 (2017).

7 E. Shani, *et al.,* Gibberellins accumulate in the elongating endodermal cells of *Arabidopsis* root. *Proc. Natl. Acad. Sci.* 110: 4834-4839 (2013).

8 M. van der Weele, H. S. Jiang, K. K. Palaniappan, V. B. Ivanov, K. Palaniappan, and T. I. Baskin. A New Algorithm for Computational Image Analysis of Deformable Motion at High Spatial and Temporal Resolution Applied to Root Growth. *Plant Physiology*, 132:1138–1148, (2003).

9 S. Li, M. Yamada, X. Han, U. Ohler, P. N. Benfey, High-Resolution Expression Map of the Arabidopsis Root Reveals Alternative Splicing and lincRNA Regulation. *Dev. Cell* **39** (2016).

10 A. R. G. Plackett, *et al.*, Analysis of the developmental roles of the arabidopsis gibberellin 20-oxidases demonstrates that GA20ox1, -2, and -3 are the dominant paralogs. *Plant Cell* (2012) https:/doi.org/10.1105/tpc.111.095109.

11 M. G. Mitchum, et al., Distinct and overlapping roles of two gibberellin 3-oxidases in Arabidopsis development. Plant J. 45, 804–818 (2006).

12 L. Willis, Y. Refahi, R. Wightman, B. Landrein, J. Teles, K.C. Huang, E.M. Meyerowitz, H. J önsson. Cell size and growth regulation in *Arabidopsis* Proc. Natl Acad. Sci. 113 (51) E8238-E8246; (2016).

13 Sugimoto-Shirasu K., Roberts K. 'Big it up': endoreduplication and cell-size control in plants. Curr. Opin. Plant Biol. (2003). **6**, 544-553.
